# Supplementary material for: Dual targeting of polyamine synthesis and uptake in diffuse intrinsic pontine gliomas
Source: Nat Commun. 2021 Feb 12;12:971. doi: 10.1038/s41467-021-20896-z (PMC7881014; doi:10.1038/s41467-021-20896-z)
Supplement: Supplementary file 1 — Supplementary Information [file 41467_2021_20896_MOESM1_ESM.pdf]

Supplementary Information

Dual targeting of polyamine synthesis and uptake in diffuse intrinsic pontine gliomas

Khan et al.

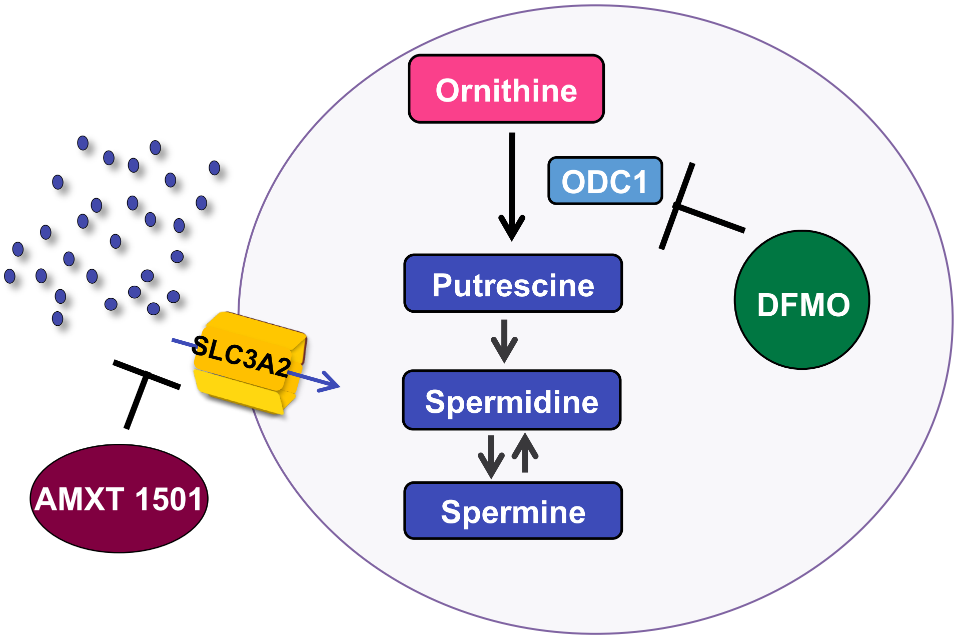

Supplementary Figure 1: An overview of the polyamine pathway. De novo synthesis of polyamines occurs naturally inside cells, from the conversion of ornithine by ODC1 into putrescine, spermidine and spermine. This synthesis can be impeded with the ODC1 inhibitor; DFMO. Import of polyamines from the external environment, either from surrounding cells or from diet, play a vital role in polyamine homeostasis. SLC3A2 has been identified as a key transporter in polyamine metabolism. Polyamine transport can be blocked by using the polyamine transport inhibitor: AMXT 1501.

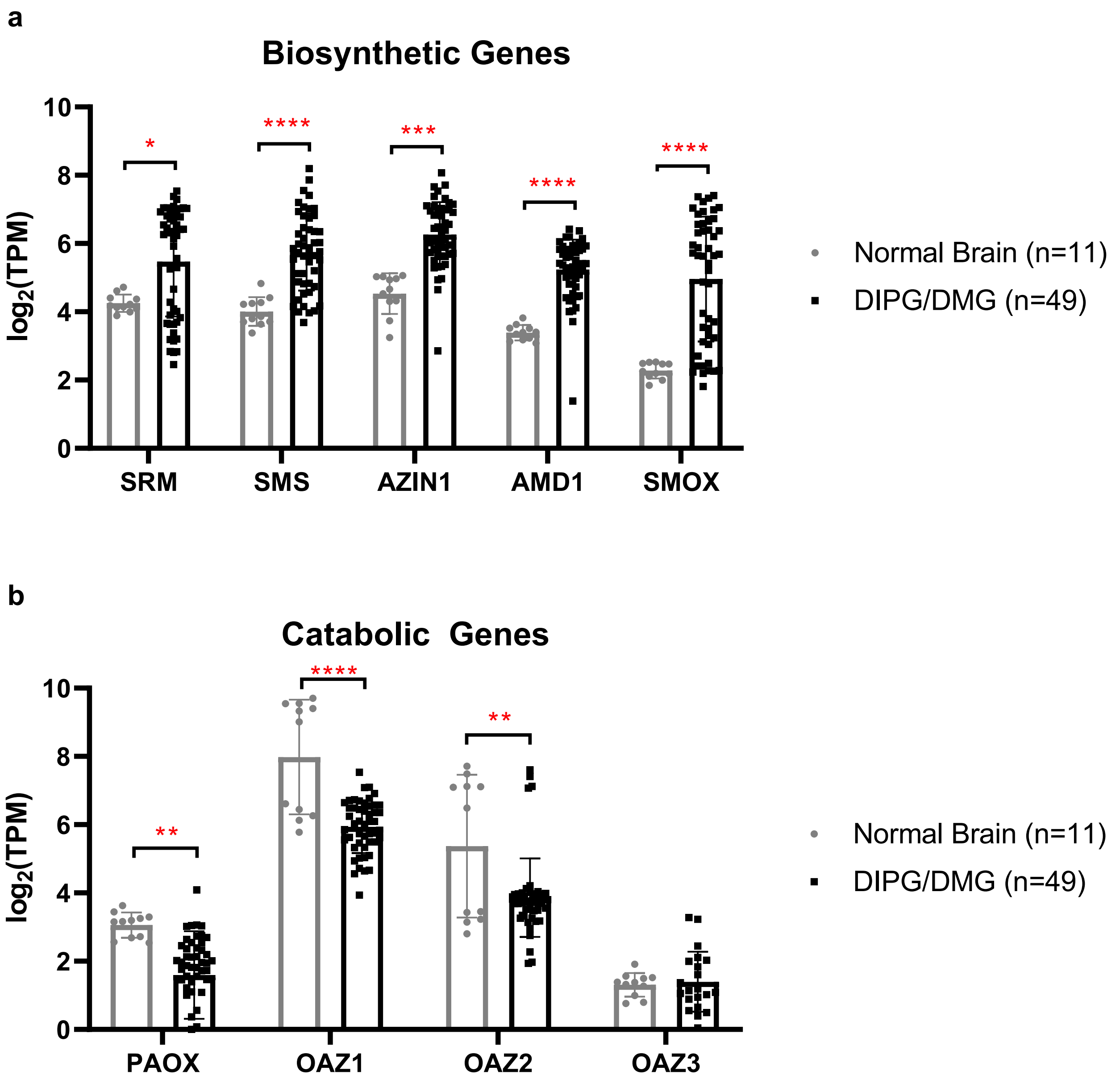

Supplementary Figure 2 : Expression of polyamine pathway genes in a DIPG tumor dataset. (a) High expression of five biosynthetic genes SRM, SMS, AZIN1, AMD1 and SMOX and **(b)** low expression of four catabolic genes PAOX, OAZ1, OAZ2 and OAZ3 in DIPG/DMG (N=49) compared to normal fetal brain (N=11). Data were obtained from ZCC cohort and McGill University[30]. Data are presented as mean values ± SD. *$p<0.05$, **$p<0.01$, ****$p<0.0001$. p-values were calculated using two-way ANOVA for normal and tumor samples. **(a)** SRM: p=0.0147, SMS: p=0.0001, AZIN1: p=0.0001, AMD1: p<0.0001, SMOX: p<0.0001. **(b)** PAOX: p=0.00021, OAZ1: p<0.0001, OAZ2: p=0.0015.

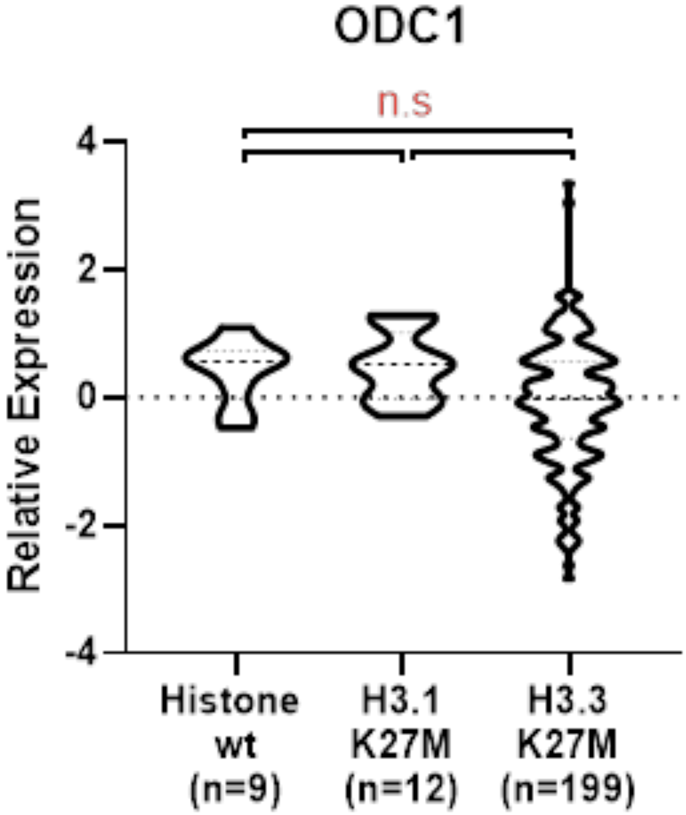

Supplementary Figure 3: ODC1 gene expression is independent of H3K27 status in DIPG; ODC1 mRNA relative expression was analysed in 220 DIPG samples from the Mackay cohort[45]. There was no statistical difference in ODC1 gene expression levels between H3K27wt, H3.1K27M and H3.3K27M samples. Statistical analysis was performed with one-way ANOVA.

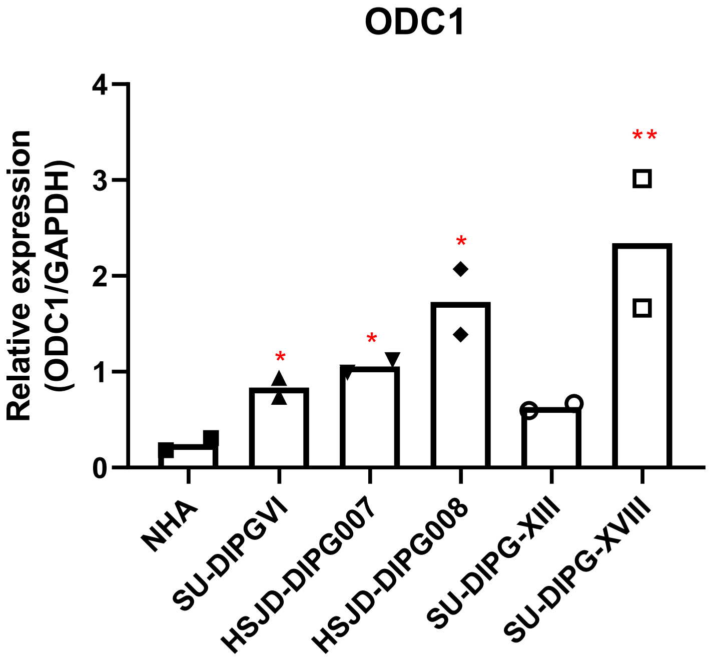

Supplementary Figure 4: ODC1 protein expression in a panel of healthy and primary DIPG cultures. Densitometric analysis of ODC1 protein bands from two independent experiments. Representative western blot is provided in Figure 1c. n=1 over two independent experiments. Data are presented as mean values ± SEM. * $p < 0.05$; ** $p < 0.005$. Statistical analysis was performed with one-way ANOVA for NHA and DIPG cell lines. NHA vs SU-DIPGVI: p=0.0339, NHA vs HSJD-DIPG007: p=0.0106, NHA vs HSJD-DIPG008: p=0.0350, NHA vs SU-DIPG-XIII: p=0.0270, NHA vs SU-DIPG-XVIII: p=0.0060.

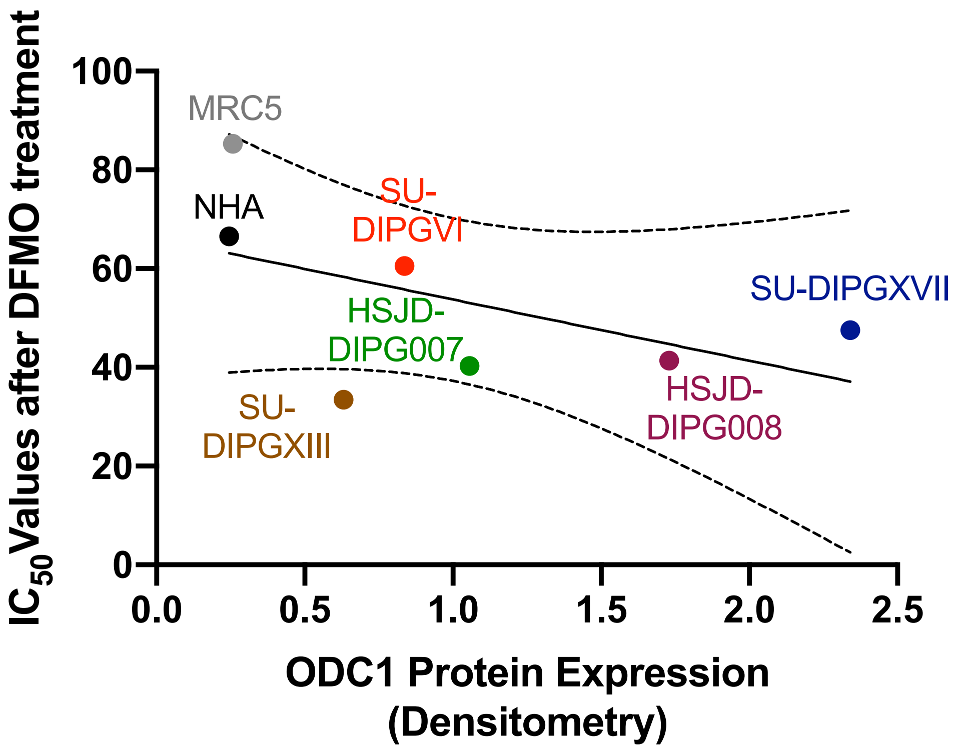

Supplementary Figure 5: Correlation between ODC1 protein levels and DFMO IC50 values; Correlation was calculated based on densitometric analysis (Figure 1c) and IC50 values (Figure 1i). There was a moderate negative correlation between ODC1 protein levels and DFMO IC50s (Pearson correlation coefficient (r) = -0.52747566).

a

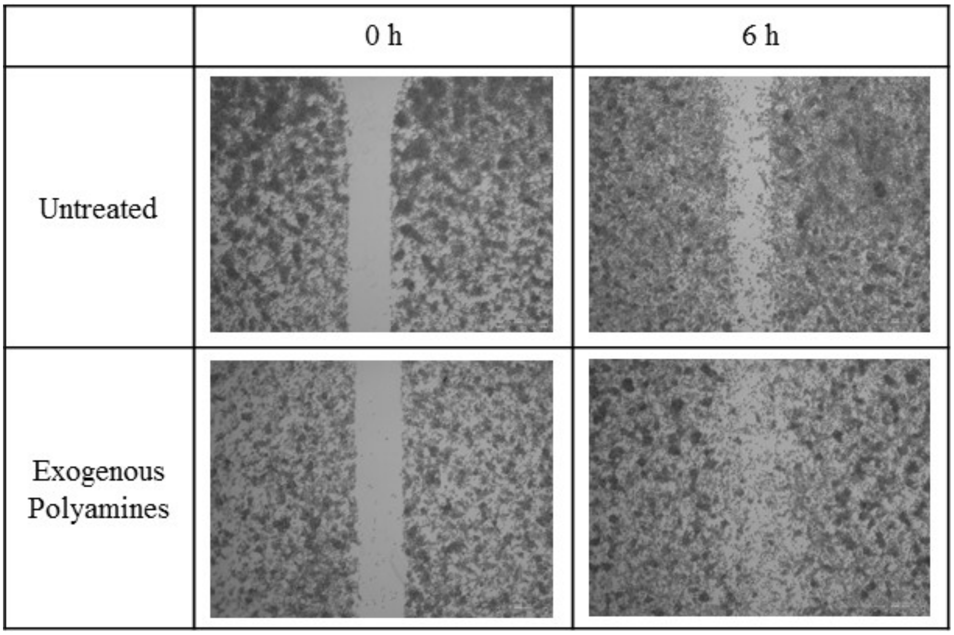

b

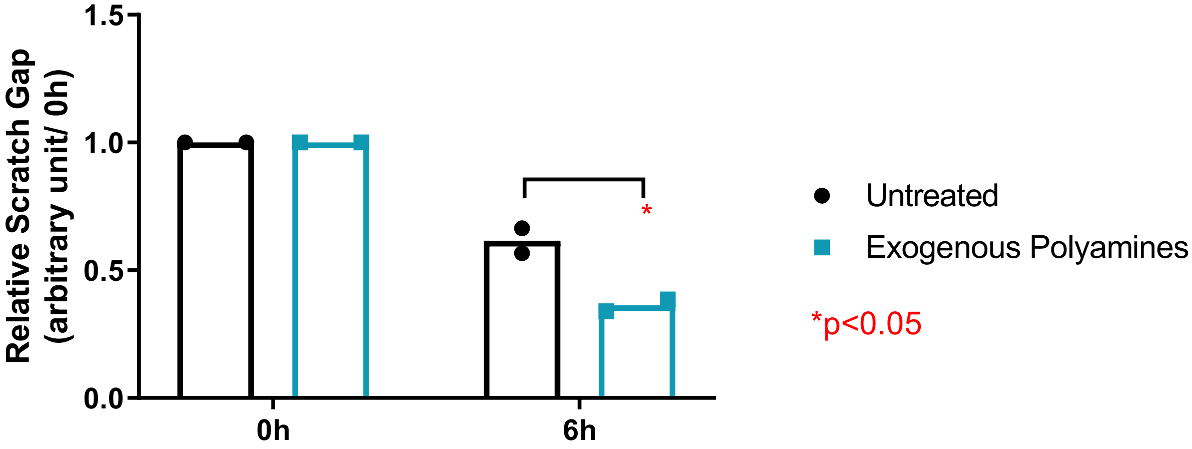

Supplementary Figure 6: Effect of additional exogenous polyamines on DIPG cell migration. **(a-b):** After addition of exogenous polyamines: putrescine, spermidine and spermine at 10uM**,** HSJD-DIPG007 cells covered distance at a faster rate than normal untreated cells Data is presented from two independent experiments. p-values were calculated using one-way ANOVA for untreated samples and samples added with exogenous polyamines ($p=0.0423$).

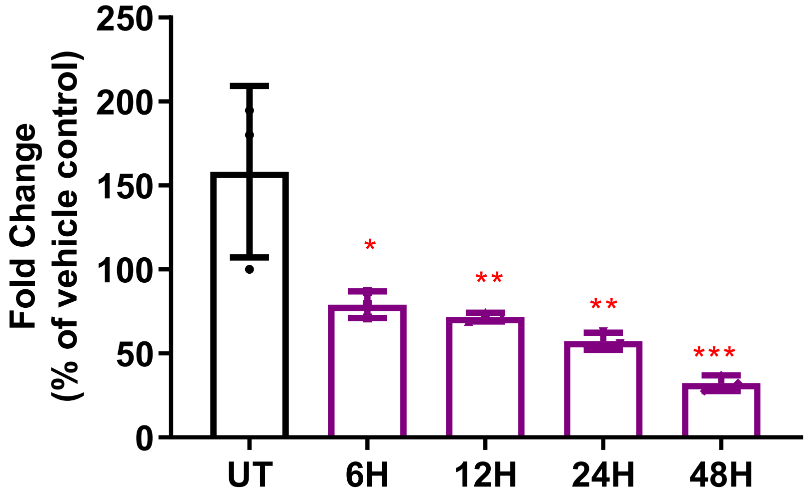

Supplementary Figure 7: Decreased ODC1 gene expression measured by RT-qPCR in HSJD-DIPG007 cells upon treatment with 40mM DFMO. Data is presented as means ± SEM of three independent experiments. *p<0.05, **p<0.01, ***p<0.001. p-values were calculated using one-way ANOVA for untreated and treated samples. UT vs 6h: p=0.013, UT vs 12h: p=0.0073, UT vs 24h: p=0.0075, UT vs 48h: p=0.0004.

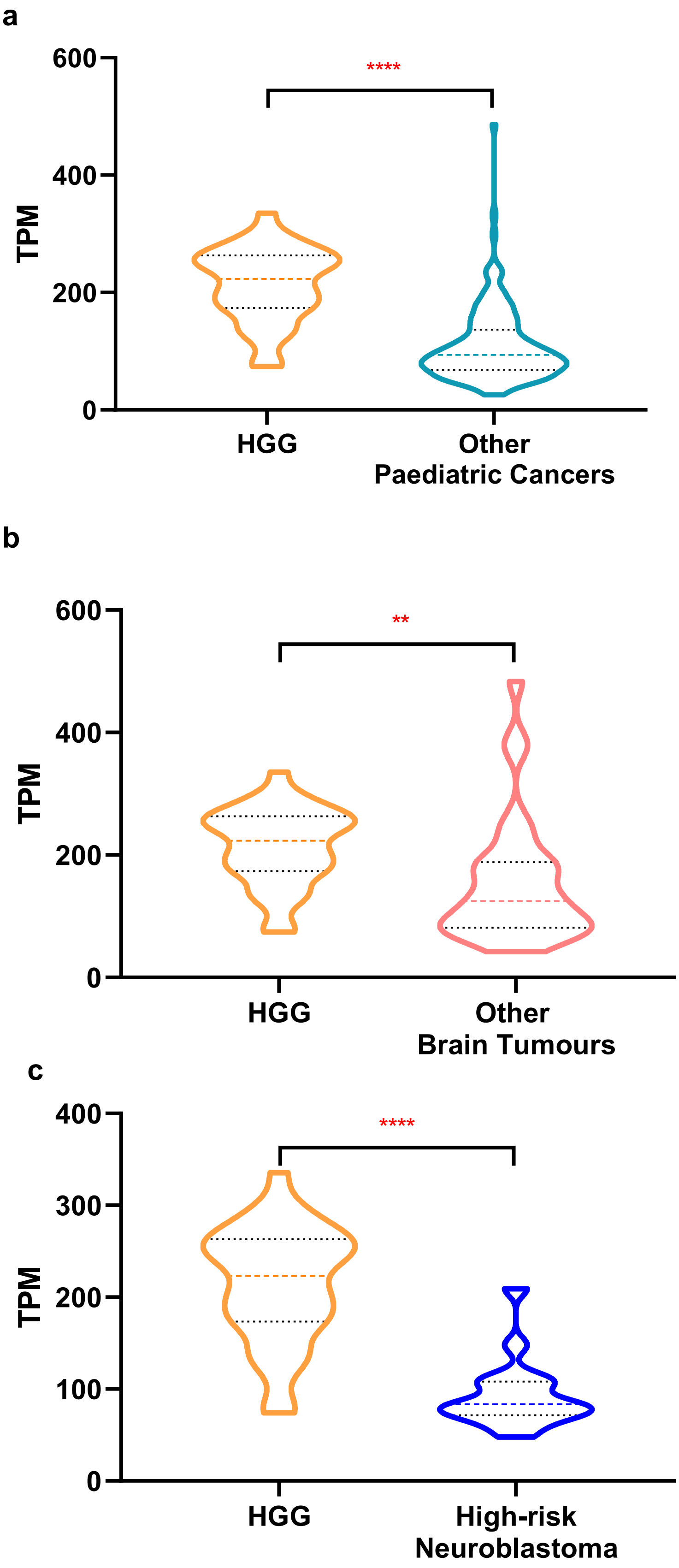

Supplementary Figure 8: Polyamine transporter expression in pediatric HGG. Examination of RNA expression levels in a cohort of high-risk childhood cancers showed that the polyamine transporter, SLC3A2, was significantly overexpressed in HGG's (n=32 biological samples) compared with **(a)** all other high-risk childhood cancers (n=148 biological samples) ($p<0.0001$), **(b)** all other high-risk brain tumors (n=34 biological samples) ($p=0.0049$) and **(c)** high-risk neuroblastomas (n=17 biological samples) ($p<0.0001$). **$p<0.01$, ****$p<0.0001$. Statistical analysis was performed with two-tailed t-test between cohorts.

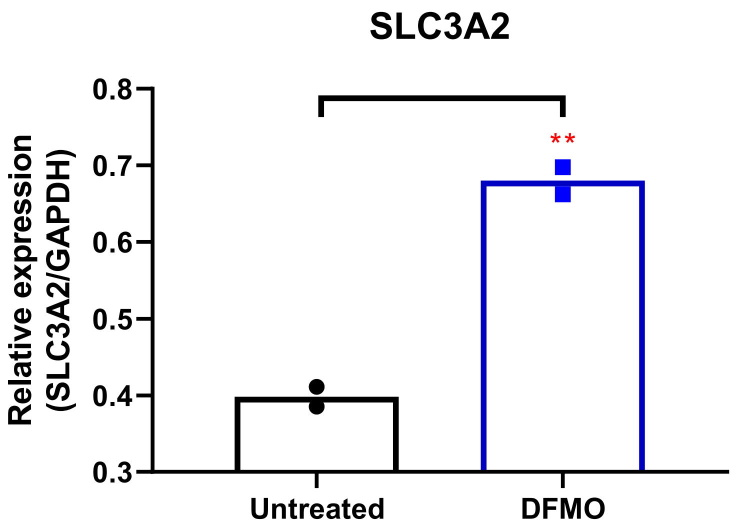

Supplementary Figure 9: SLC3A2 protein expression increases following treatment with DFMO. Densitometric analysis of SLC3A2 protein bands in DFMO treated DIPG cells from two independent experiments. Representative western blot is provided in Figure 2d. **p<0.005. Statistical analysis was performed with two-tailed t-test, p=0.0060.

a

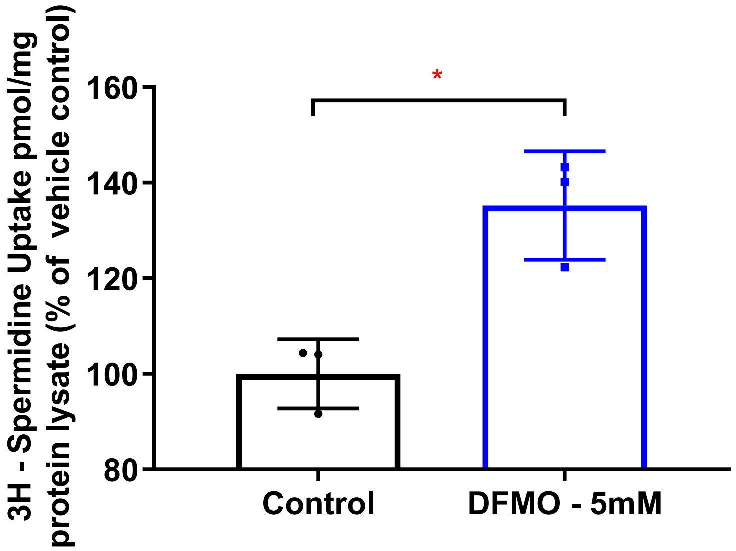

b

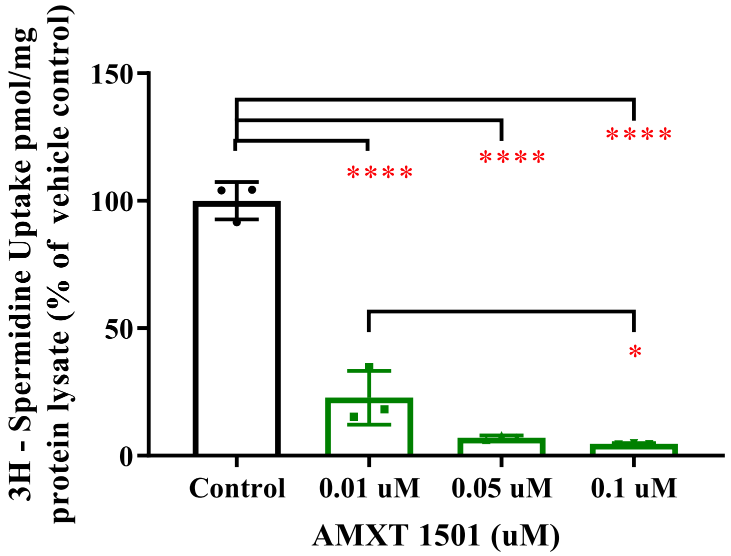

Supplementary Figure 10: Polyamine uptake effects in SU-DIPGVI cells following DFMO and AMXT 1501 treatment. Uptake of radiolabeled spermidine **(a)** increased following 5mM DFMO treatment ($p=0.0105$) and **(b)** decreased with AMXT 1501 treatment in SU-DIPGVI cells. Data is presented as means ± SEM of three independent experiments. *$p<0.05$, ****$p<0.0001$. Statistical analysis was performed with **(a)** two-tailed t-test and **(b)** one-way ANOVA between treatment cohorts. **(b)** Control vs 0.01: $p<0.0001$, Control vs 0.05: $p<0.0001$, Control vs 0.1: $p<0.0001$, 0.01 vs 0.1: $p=0.0351$.

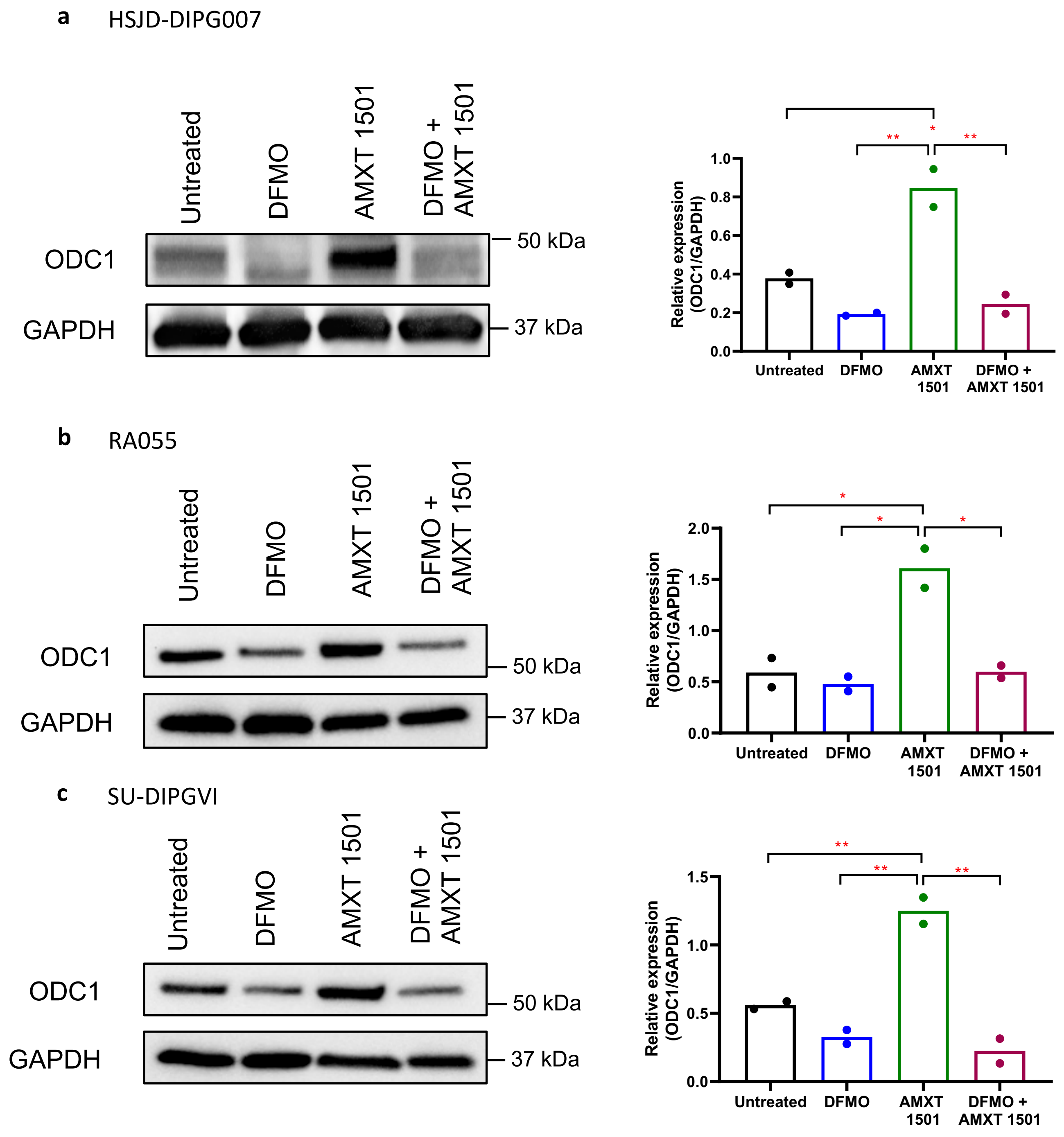

Supplementary Figure 11: ODC1 protein expression in DIPG cells after 24h of treatment with DFMO (40mM) and AMXT 1501 (5uM). In **(a)** HSJD-DIPG007 cells, **(b)** RA055 cells and **(c)** SU-DIPGVI cells, treatment with DFMO resulted in decreased ODC1 protein levels. Polyamine transport inhibition via AMXT 1501 led to an increase in ODC1 expression. The combination treatment resulted in decreased ODC1 expression. Densitometric analysis of ODC1 protein bands from two independent experiments. $^*p<0.05$. Statistical analysis was performed by using one-way ANOVA between treatment groups. **(a)** Untreated vs AMXT 1501: p=0.0149, DFMO vs AMXT 1501: p=0.0044, AMXT 1501 vs DFMO/AMXT 1501: p=0.0059. **(b)** Untreated vs AMXT 1501: p=0.0167, DFMO vs AMXT 1501: p=0.0116, AMXT 1501 vs DFMO/AMXT 1501: p=0.0172. **(c)** Untreated vs AMXT 1501: p=0.0086, DFMO vs AMXT 1501: p=0.0029, AMXT 1501 vs DFMO/AMXT 1501: p=0.0019.

HSJD-DIPG007

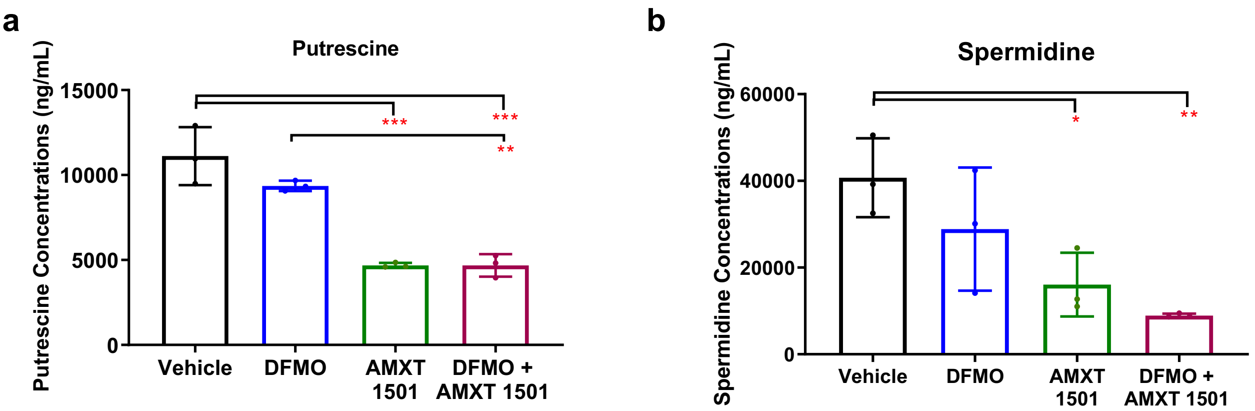

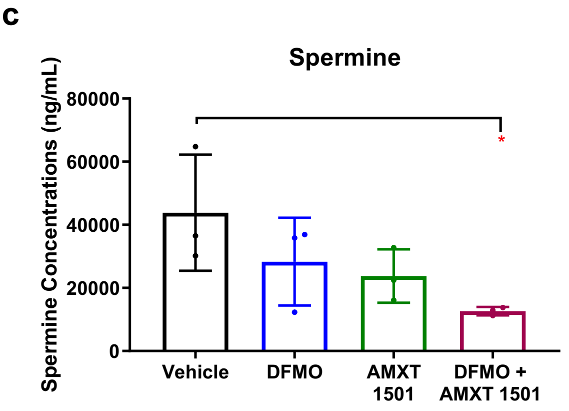

Polyamine	Synergy Index	
Putrescine	0.89	Synergistic
Spermidine	0.71	Synergistic
Spermine	0.86	Synergistic

Supplementary Figure 12: Polyamine levels in HSJD-DIPG007 cells following DFMO and AMXT 1501 treatments: (a) Putrescine, **(b)** Spermidine and **(c)** Spermine levels in HSJD-DIPG007 cells treated with DFMO and AMXT 1501 alone and in combination. Data is presented as means ± SEM of three independent experiments. *p<0.05, **p<0.01, ***p<0.0001. Statistical analysis was performed by one-way ANOVA for untreated and treated samples. Synergy calculated using the Chou-Talalay method. **(a)** Vehicle vs AMXT 1501: p=0.0001, Vehicle vs DFMO/AMXT 1501: p=0.0001, DFMO vs DFMO/AMXT 1501: p=0.0019. **(b)** Vehicle vs AMXT 1501: p=0.0445, Vehicle vs DFMO/AMXT 1501: p=0.01. **(c)** Vehicle vs DFMO/AMXT 1501: p=0.0473.

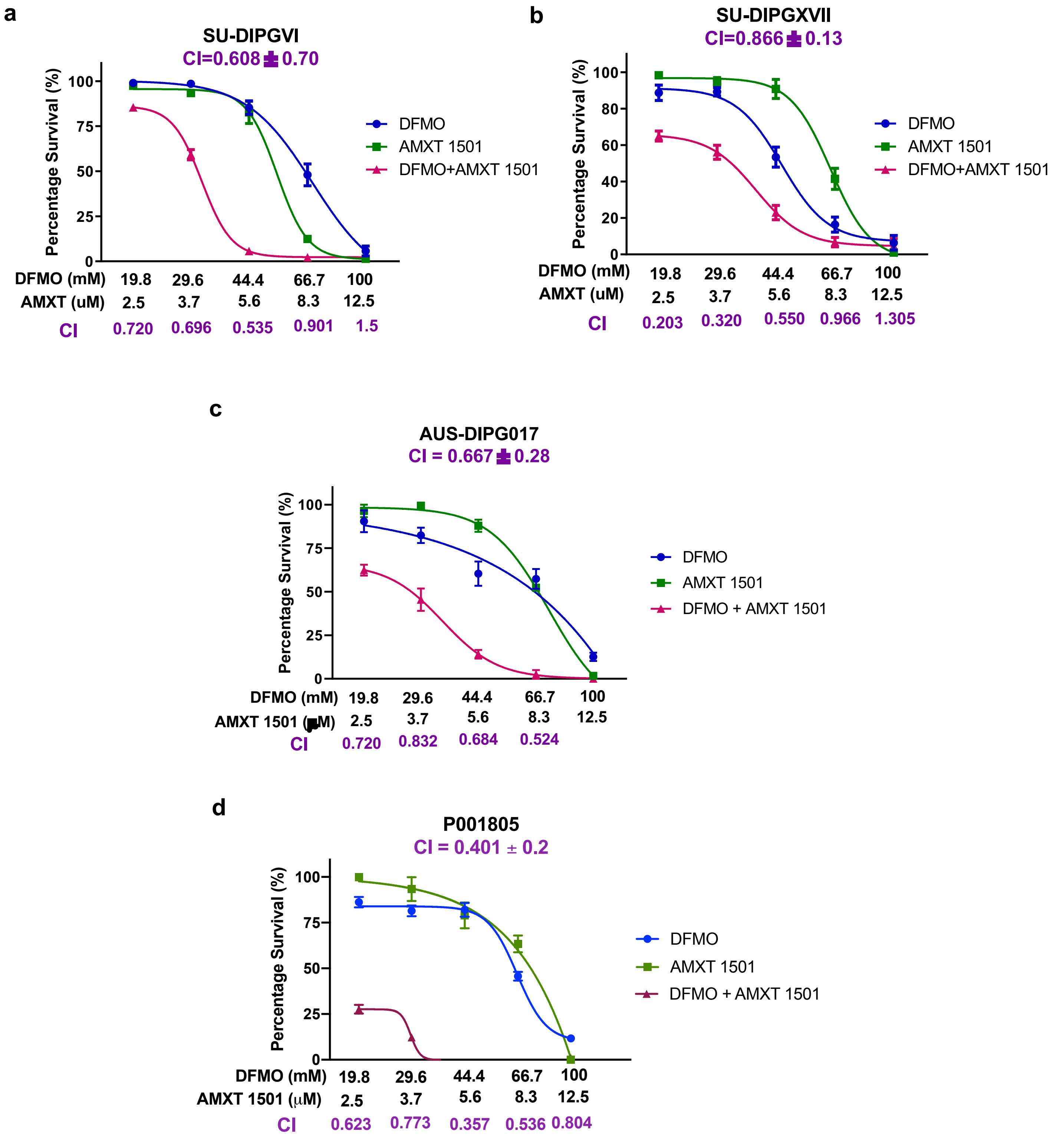

Supplementary Figure 13: Combined inhibition of polyamine synthesis and uptake in DIPG/DMG. AMXT 1501 plus DFMO treatment is synergistic in DIPG cultures **(a)** SU-DIPGVI, **(b)** SU-DIPGXVII and in thalamic midline glioma cultures: **(c)** AUS-DIPG017 **(d)** P001805. Data is presented as means ± SEM of three independent experiments. CI values were calculated using Calcusyn.

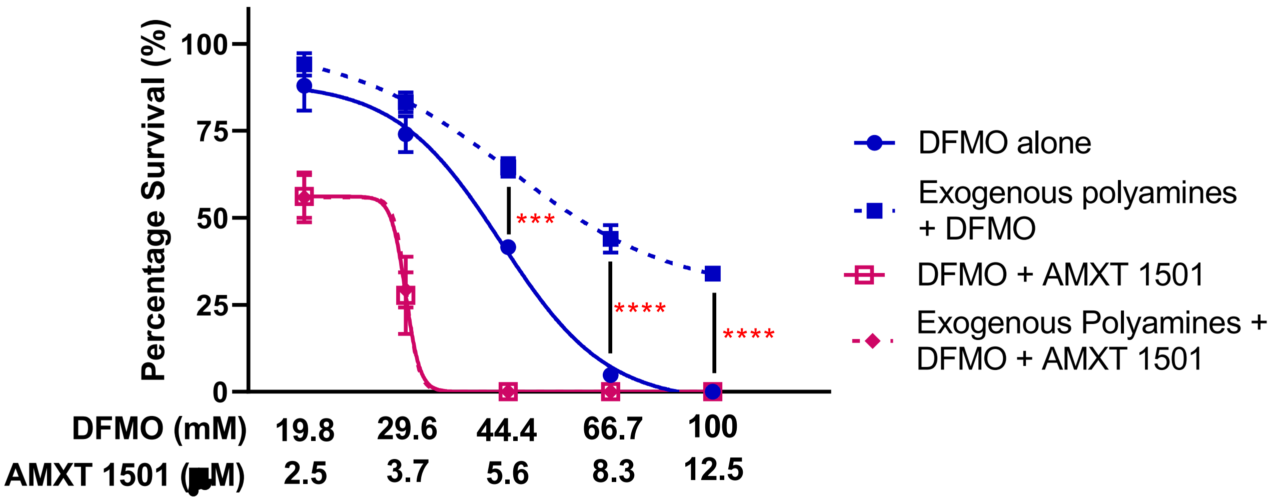

Supplementary Figure 14: Exogenous polyamines are ineffective following treatment with DFMO and combination of DFMO with AMXT 1501. Exogenous addition of polyamines reduced the cytotoxic potential of DFMO, however, have no effect following treatment with AMXT 1501. Data is presented as means ± SEM of three independent experiments. ***$p<0.001$, ****$p<0.0001$. Statistical analysis was performed with two-tailed t-tests. DFMO alone vs Exogenous polyamines + DFMO: 44.4mM: $p=0.0001$, 66mM: $p<0.0001$, 100mM: $p<0.0001$.

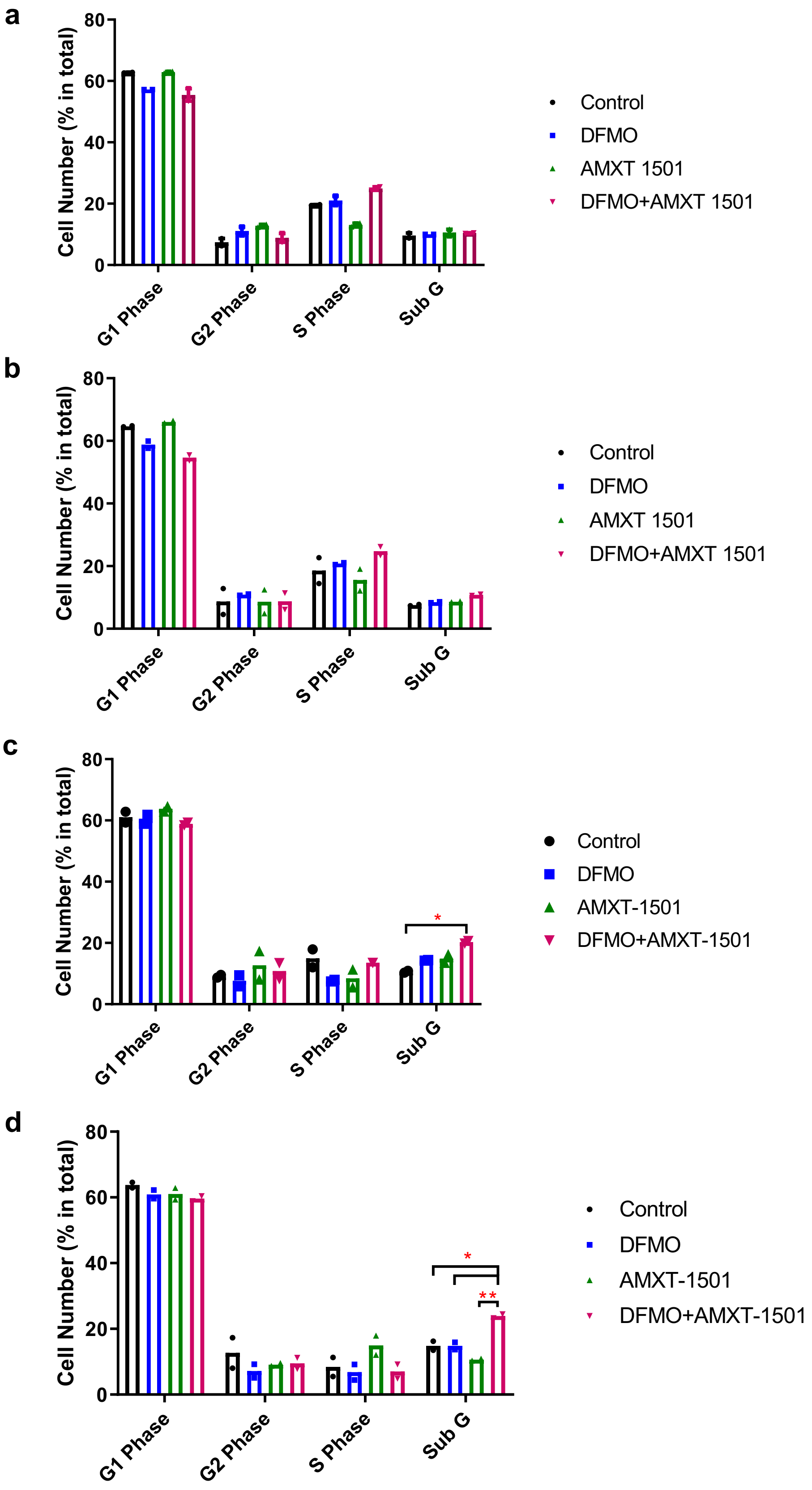

Supplementary Figure 15: **Effect of DFMO and AMXT 1501 on cell cycle in HSJD-DIPG007 cells.** 40mM DFMO and 5uM AMXT 1501 exhibited no significant change in any stage of the cell cycle at either **(a)** 18h, **(b)** 21h, or **(c)** 24h post treatment. There was a significant increase in the sub G1 population after **(d)** 48 hours. Data is presented from two independent experiments. $p<0.05$, ** $p<0.01$, Statistical analysis was performed using one-way ANOVA between treatment groups. **(c)** Control vs DFMO/AMXT 1501: $p=0.0156$. **(d)** Control vs DFMO/AMXT 1501: $p=0.0279$, DFMO vs DFMO/AMXT 1501: $p=0.0269$, AMXT 1501 vs DFMO/AMXT 1501: $p=0.0014$.

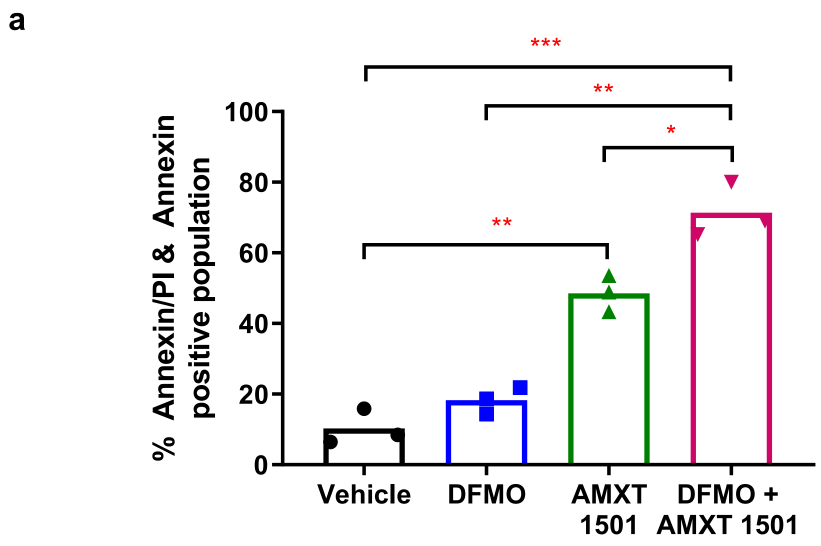

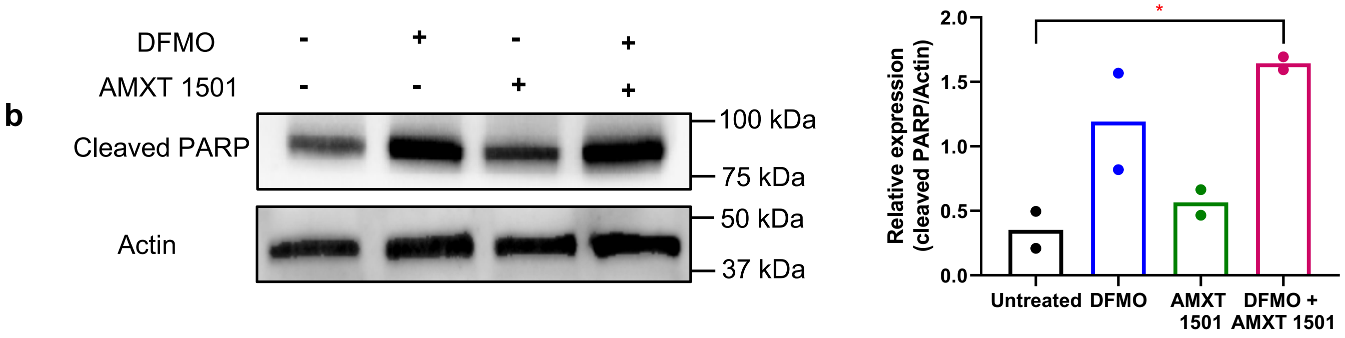

Supplementary Figure 16: Apoptotic effects of the DFMO and AMXT 1501 combination in SU-DIPGVI cells. (a): Annexin/PI staining of SU-DIPGVI cells treated with DFMO (50mM), AMXT 1501 (6uM), or combination of both agents for 24h. **(b)**: Apoptotic effects of the DFMO and AMXT 1501 combination as determined by cleaved PARP protein expression. Representative blot from two independent experiments. Data is presented from two independent experiments. *p<0.05, **p<0.01, ***p<0.001. Statistical analysis was performed by one-way ANOVA for untreated and treated samples. **(a)** Vehicle vs AMXT 1501: p=0.0040, Vehicle vs DFMO/AMXT 1501: p=0.0005, DFMO vs DFMO/AMXT 1501: p=0.0009, AMXT 1501 vs DFMO/AMXT 1501: p=0.0122. **(b)** Vehicle vs DFMO/AMXT 1501: p=0.0392.

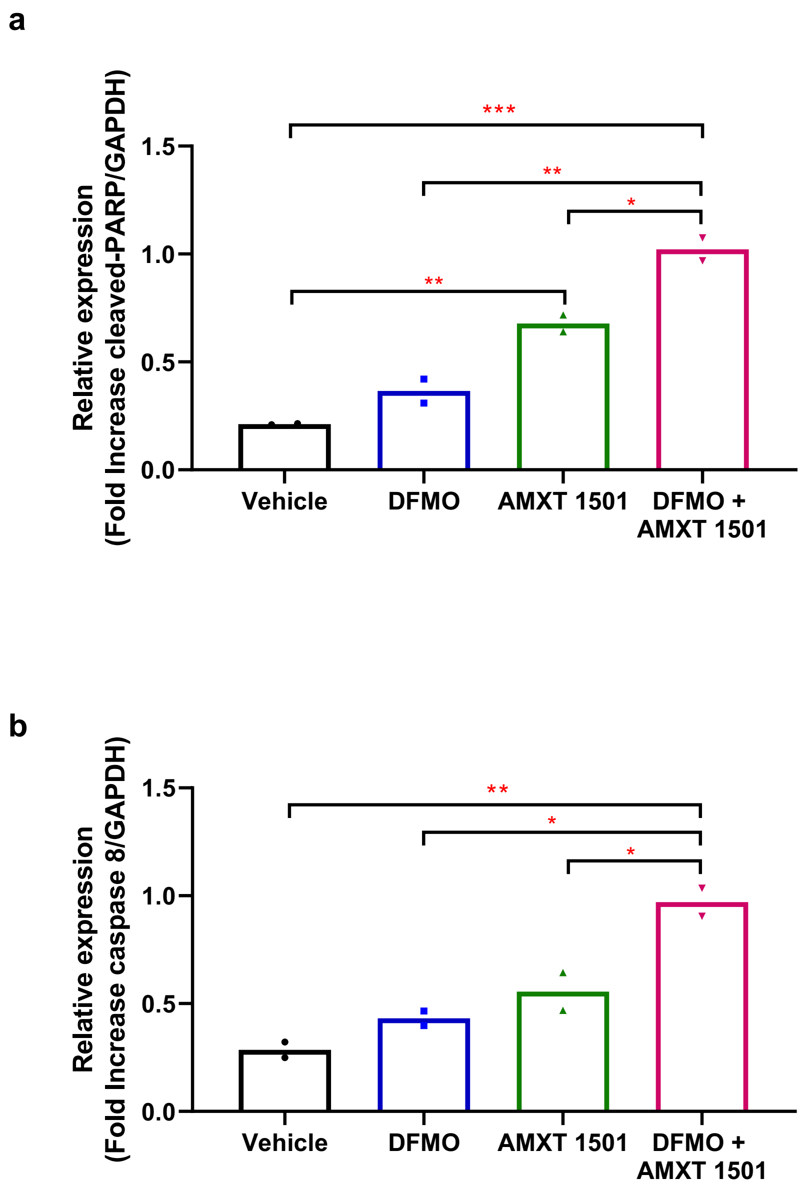

Supplementary Figure 17: Effect of polyamine pathway inhibition on apoptosis markers. Densitometric analysis of **(a)** cleaved parp and **(b)** caspase 8 protein bands in DFMO, AMXT 1501 and combination treated DIPG cells. Representative western blot is given in Figure 4e. Data are presented as mean values ± SEM from two independent experiments. n=1 over two independent experiments. *$p<0.05$, **$p<0.01$, ***$p<0.001$. Statistical analysis was performed with one-way ANOVA. **(a)** Vehicle vs AMXT 1501: p=0.0036, Vehicle vs DFMO/AMXT 1501: p=0.0004, DFMO vs DFMO/AMXT 1501: p=0.0015, AMXT 1501 vs DFMO/AMXT 1501: p=0.0164. **(b)** Vehicle vs DFMO/AMXT 1501: p=0.0029, DFMO vs DFMO/AMXT 1501: p=0.0107, AMXT 1501 vs DFMO/AMXT 1501: p=0.0269.

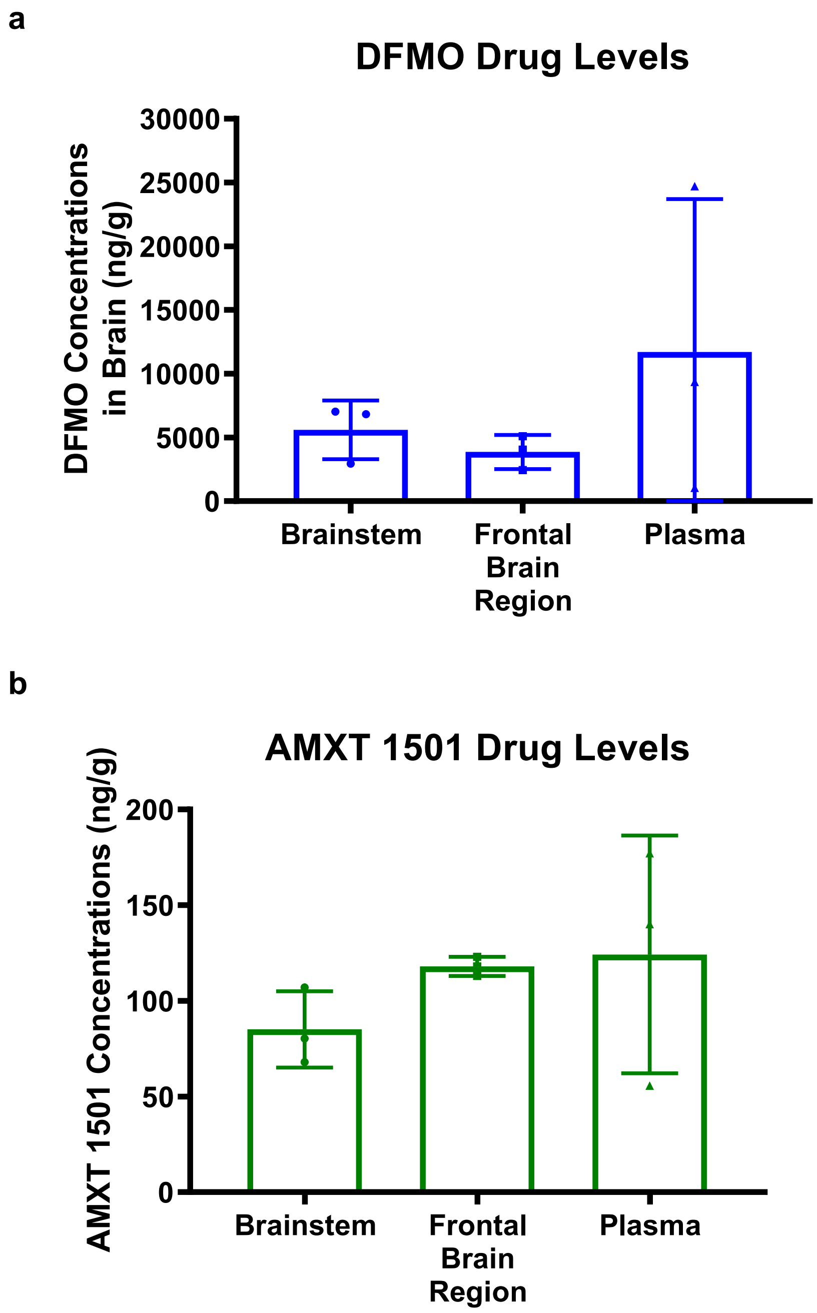

Supplementary Figure 18: Drug concentrations of **(a)** 1% DFMO and **(b)** 2.5mg/kg AMXT 1501, post 1 week of treatment in the brainstem region, frontal brain region and in the plasma of Balb/C Nude mice injected with SU-DIPGVI tumors. Samples were collected from three mice from each cohort. Data is presented as means ± SEM.

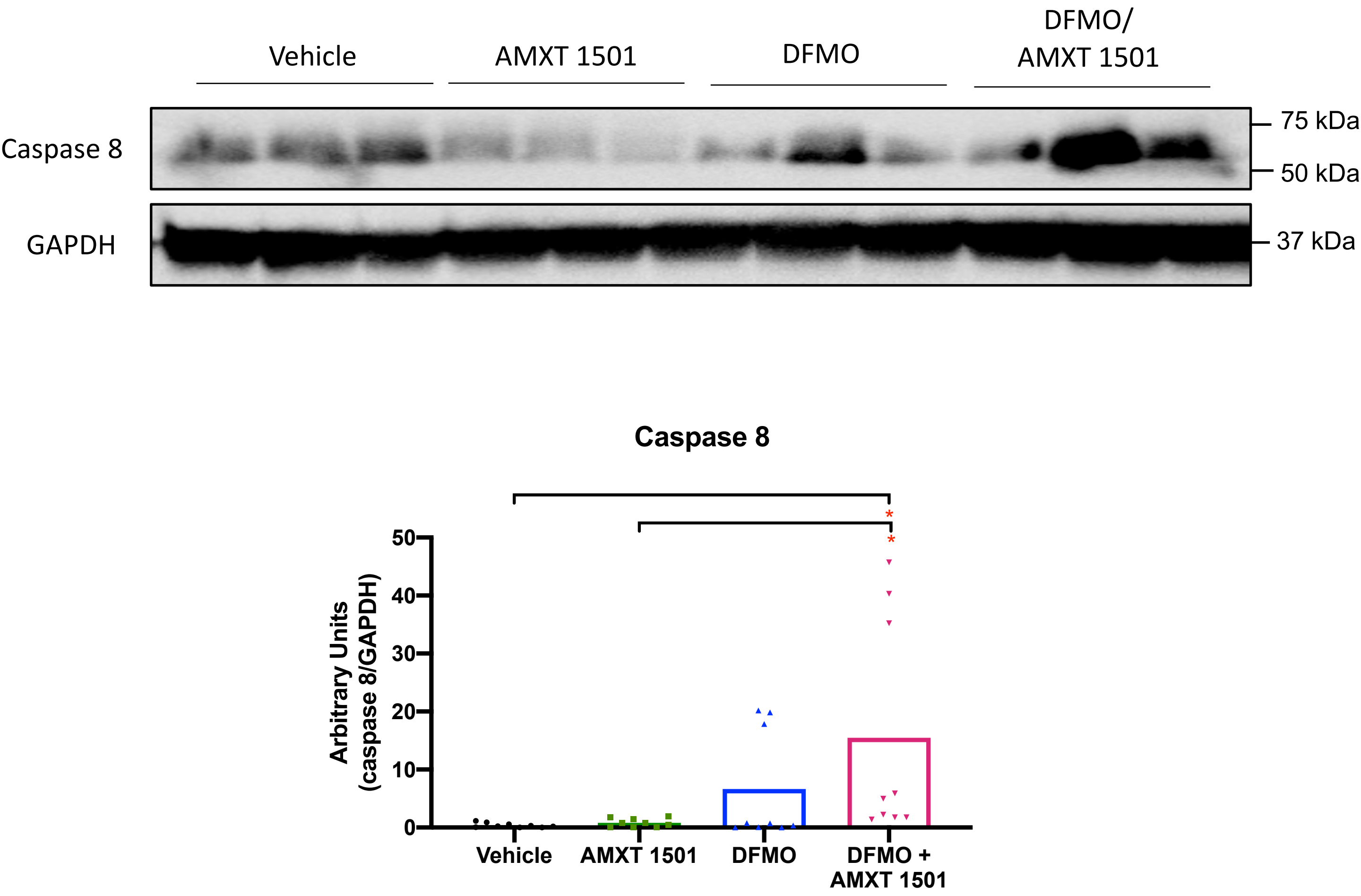

Supplementary Figure 19: Combination of DFMO with AMXT 1501 increases caspase 8 protein levels. Twelve HSJD-DIPG007 xenografted animals were treated with polyamine inhibitors for 4 weeks. Western blot analysis of protein extracted from each tumor (3 per group) showed increased protein levels of caspase 8 in combination animals treated compared to treatment with single agents. Western blot is representative of two independent replicates. *p<0.05. Statistics was calculated using one-way ANOVA. Vehicle vs DFMO/AMXT 1501: p=0.0243, AMXT 1501 vs DFMO/AMXT 1501: p=0.0296.

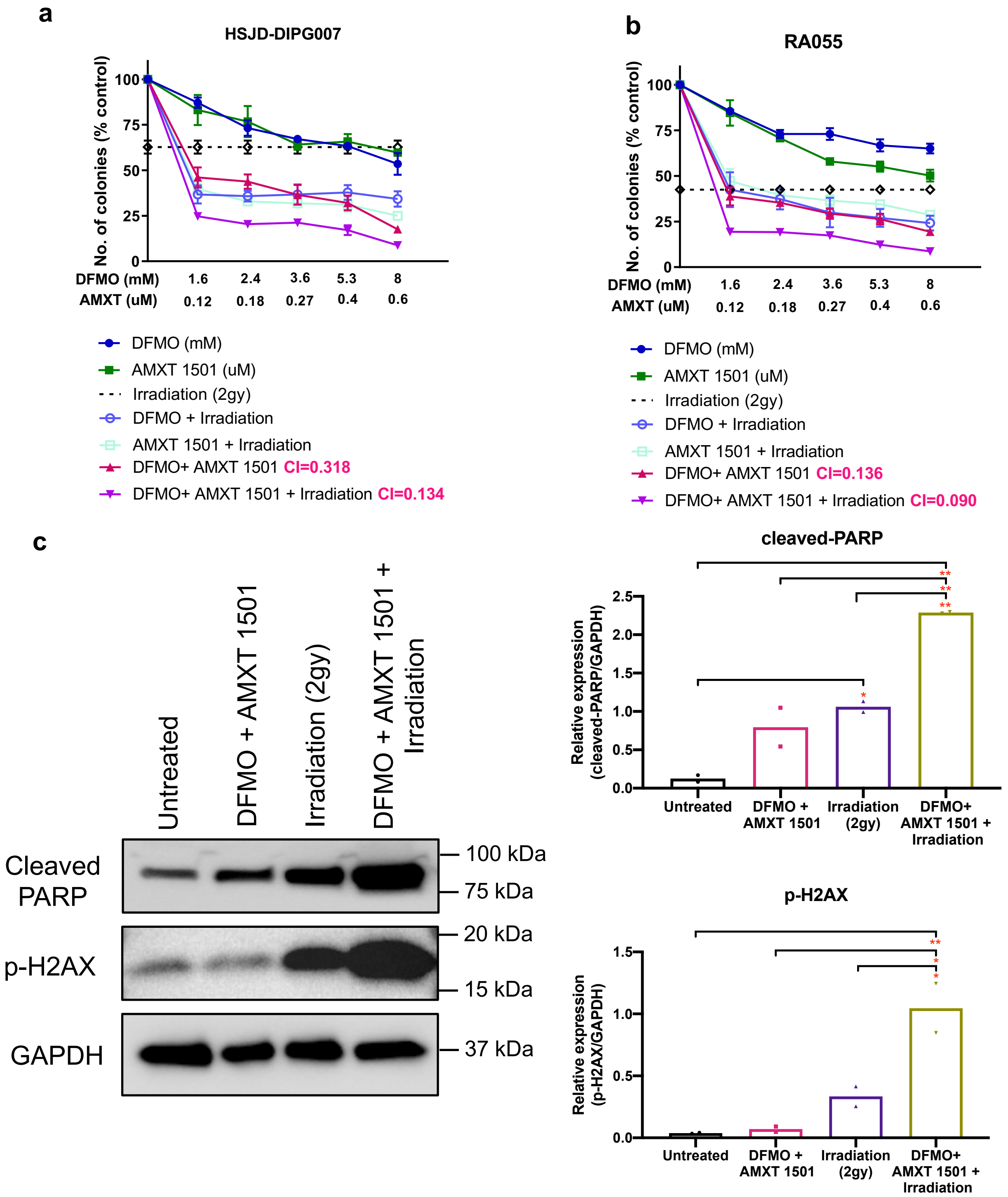

Supplementary Figure 20: Combination of irradiation with polyamine inhibitors reduces the clonogenic potential of DIPG cells. DIPG cells HSJD-DIPG007 **(a)** and RA055 **(b)** exhibit reduced colony formation on soft agar following treatment with various concentrations of DFMO, AMXT 1501, combination and 2Gy irradiation. Data is presented as means ± SD from three independent experiments. **(c)** Combination of polyamine depletion therapy with irradiation increases cleaved parp and p-H2AX protein levels in RA055 cells. Representative western blot from 2 independent experiments. *p<0.05, **p<0.01 Statistical analysis was performed by one-way ANOVA for treated and untreated samples. cleaved-parp: Untreated vs Irradiation: p=0.0255, Untreated vs DFMO/AMXT1501/Irradiation: p=0.0011, DFMO vs DFMO/AMXT1501/Irradiation: p=0.0047, Irradiation vs DFMO/AMXT1501/Irradiation: p=0.0098. p-H2AX: Untreated vs DFMO/AMXT1501/Irradiation: p=0.0093, DFMO vs DFMO/AMXT1501/Irradiation: p=0.0105, Irradiation vs DFMO/AMXT1501/Irradiation: p=0.0319.

a

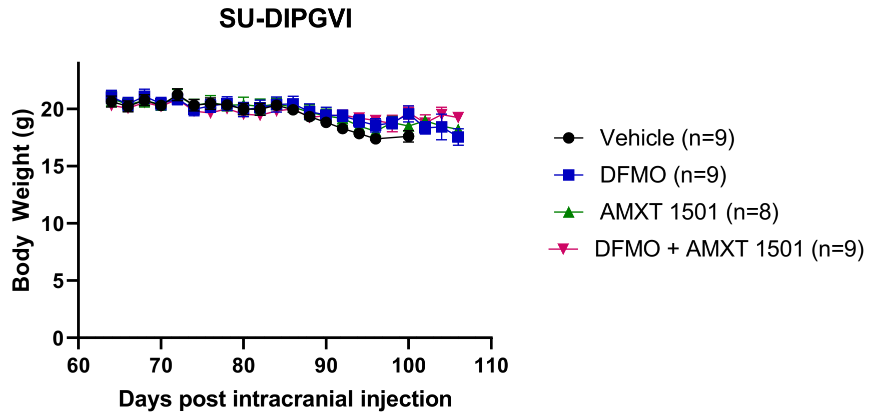

b

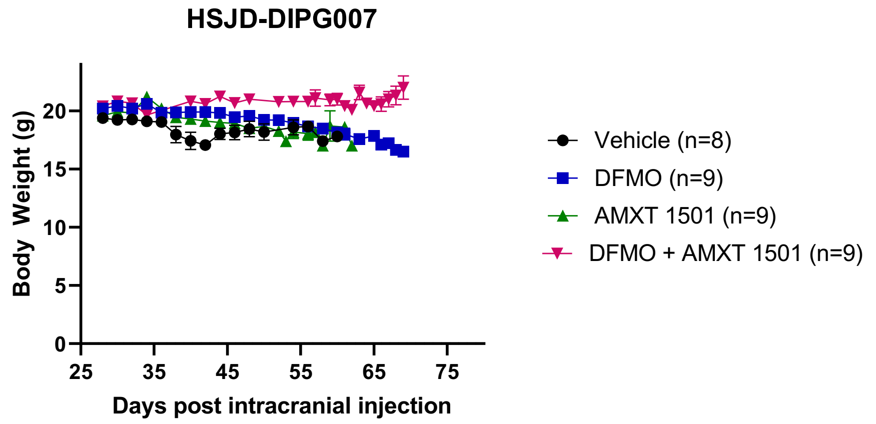

Supplementary Figure 21: DIPG bearing mice treated with DFMO and/or AMXT 1501 maintain healthy weights. SU-DIPGVI-LUC **(a)** and HSJD-DIPG007 **(b)** display stable weights during treatment with single agents and combination of DFMO with AMXT 1501. Data are presented as mean values ± SEM. SU-DIPGVI: Vehicle (n=9 mice), DFMO (n=9 mice), AMXT 1501 (n=8 mice), DFMO + AMXT 1501 (n=9 mice). HSJD-DIPG007: Vehicle (n=8 mice), DFMO (n=9 mice), AMXT 1501 (n=9 mice), DFMO + AMXT 1501 (n=9 mice).

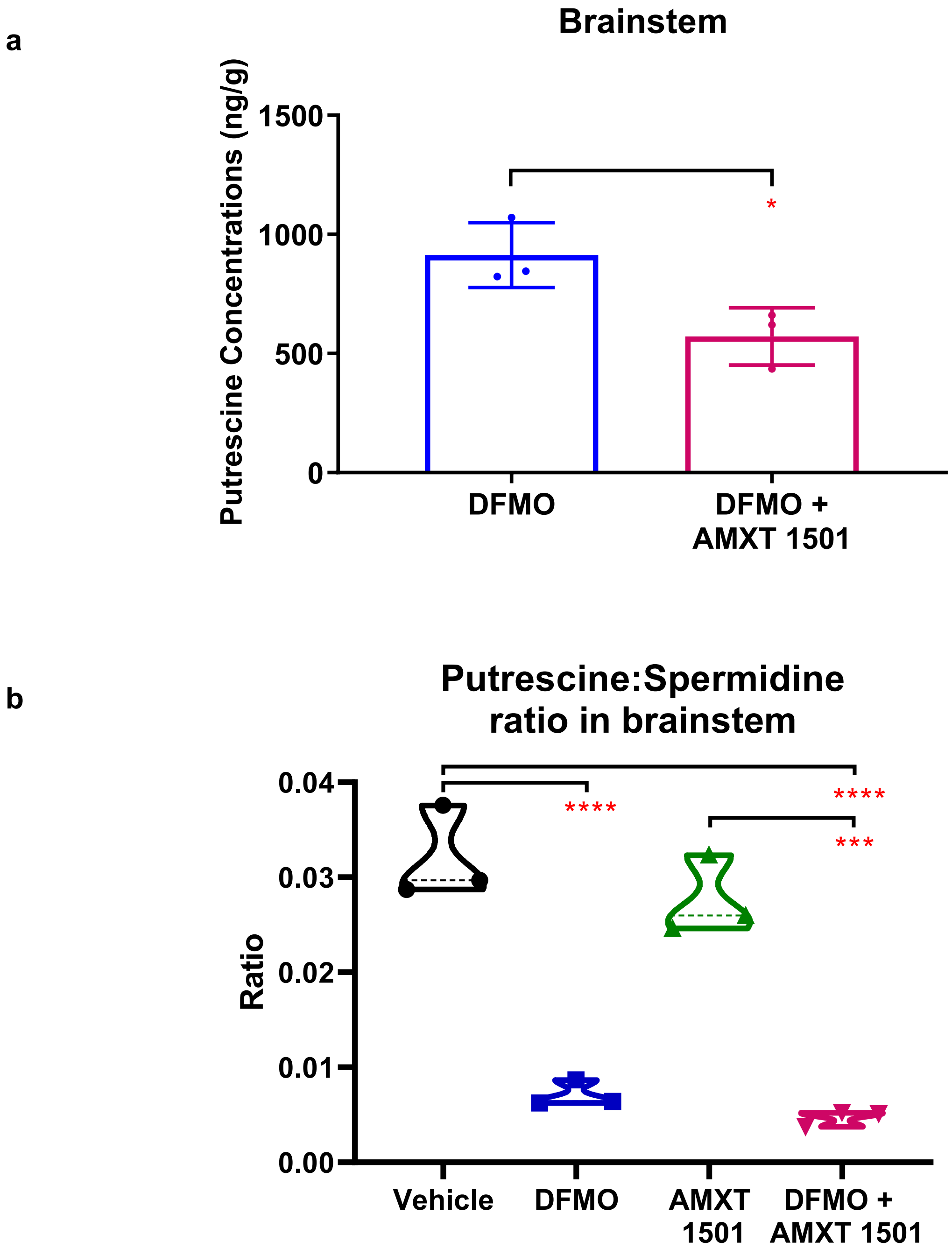

Supplementary Figure 22: Polyamine levels in animals treated with polyamine inhibitors. (a) Putrescine levels in the brainstem and (b) Putrescine to spermidine (put:spd) ratios following treatment with DFMO and DFMO/AMXT 1501**.** Data are presented as mean values ± SEM from brain samples collected from n=3 mice in each cohort. *$p<0.05$, ***$p<0.001$, ****$p<0.0001$. Statistical analysis was performed with (a) unpaired t-test and (b) one-way ANOVA between treatment cohorts. **(a)** DFMO vs DFMO/AMXT 1501: $p=0.0156$. **(b)** Vehicle vs DFMO: $p<0.0001$, Vehicle vs DFMO/AMXT 1501: $p<0.0001$, AMXT 1501 vs DFMO/AMXT 1501: $p=0.0001$.

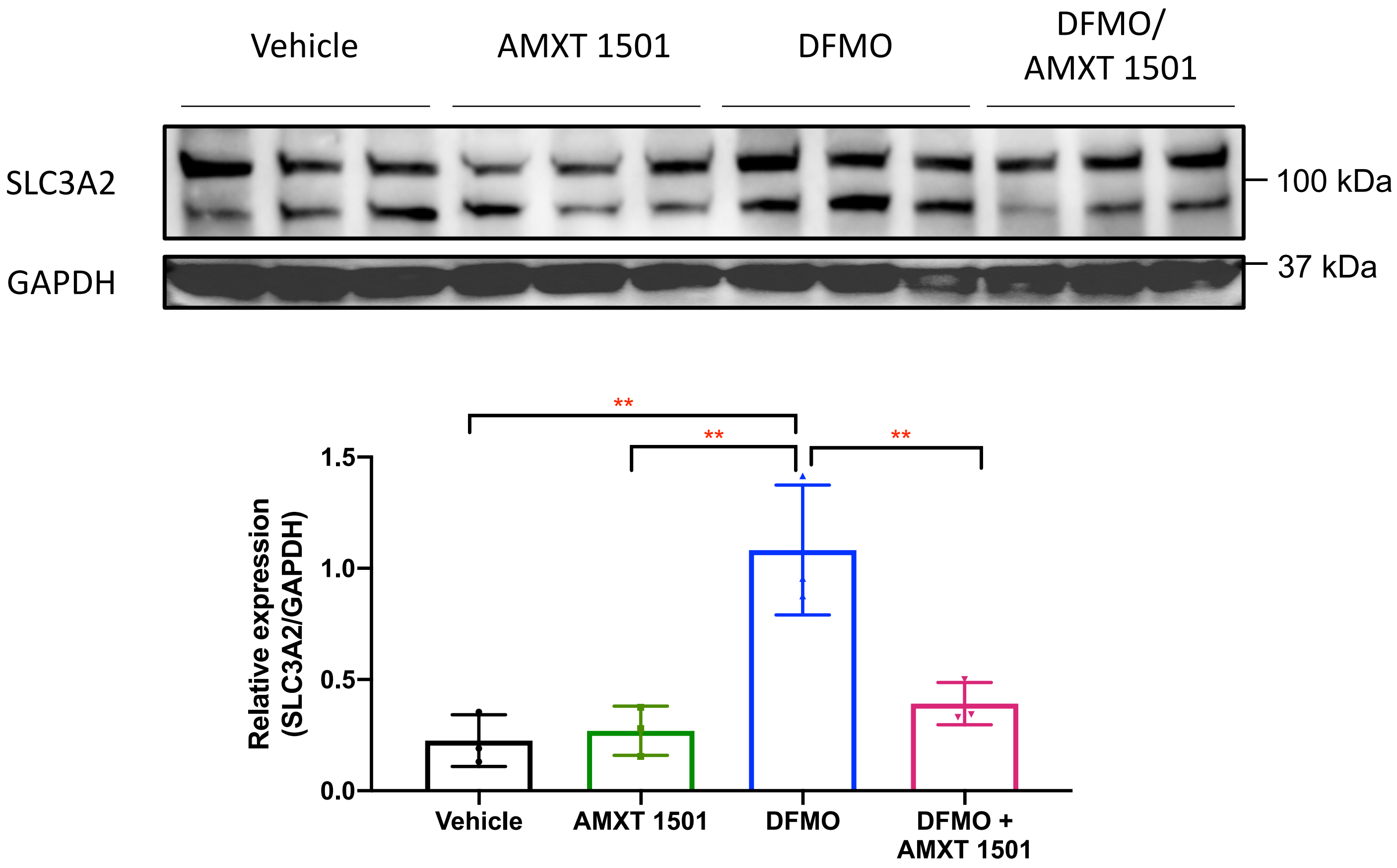

Supplementary Figure 23: Effect of DFMO/AMXT 1501 combination on SLC3A2 protein levels in vivo; SU-DIPGVI-LUC orthotopic animal models were treated with DFMO, AMXT1501 or combination for 1 week. Brains were harvested and analysed by western blotting for the protein levels of SLC3A2. Samples were collected from three mice from each cohort. Densitometric analysis showed higher levels of SLC3A2 in DFMO treated animals. Data is presented as means ± SEM. **$p<0.01$. Statistical analysis was performed by one-way ANOVA for untreated and treated samples. Vehicle vs DFMO: $p=0.0014$, AMXT 1501 vs DFMO: $p=0.0019$, DFMO vs DFMO/AMXT 1501: $p=0.0053$.

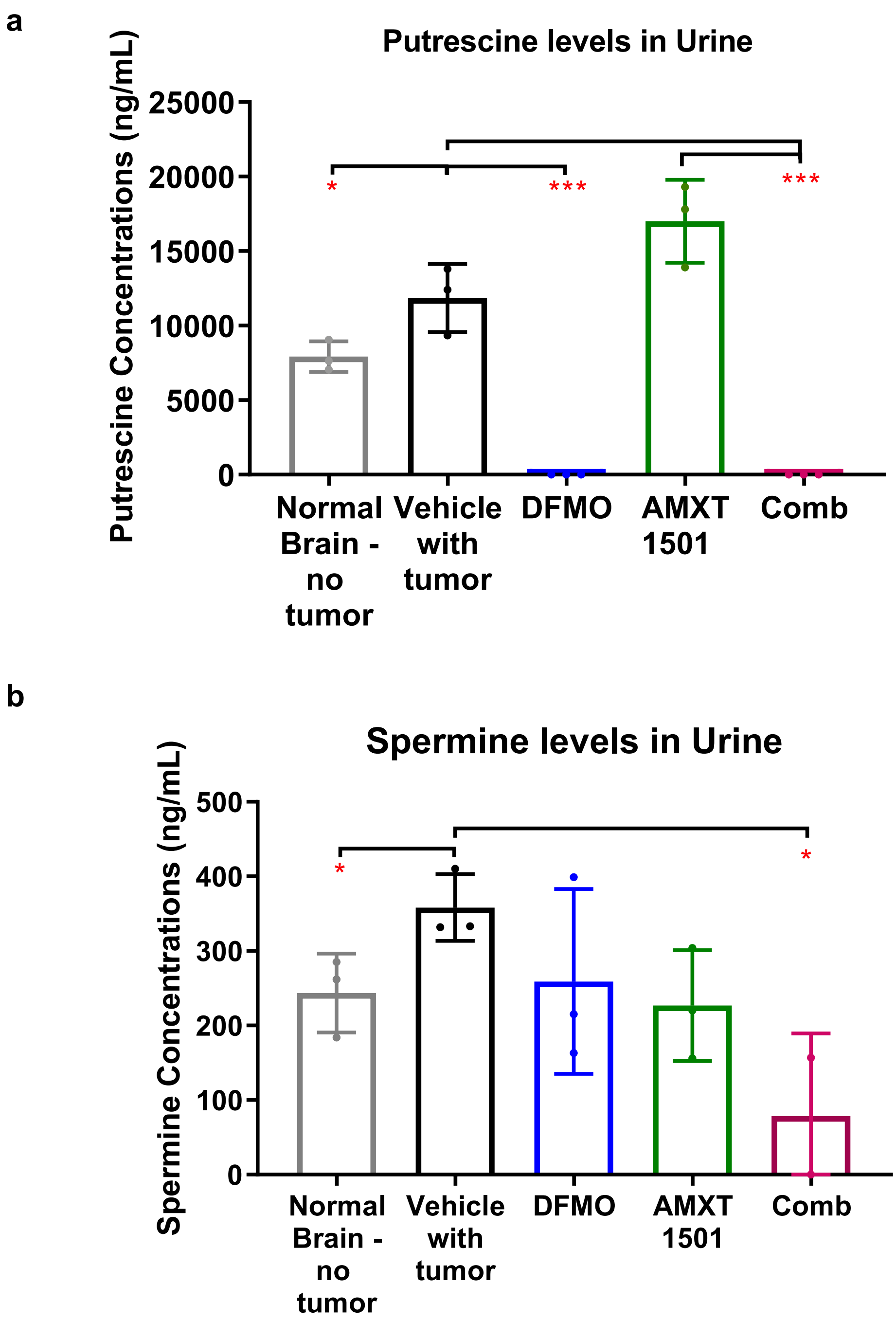

Supplementary Figure 24: Urinary polyamine concentrations of Balb/C Nude mice. (a) Putrescine and **(b)** spermine levels were significantly increased in urine of mice with tumors, compared to mice without tumors. The combination treatment of DFMO and AMXT 1501 significantly decreased polyamine concentrations compared to untreated and single agents. Samples were collected from three mice from each cohort. Data is presented as means ± SEM. *p<0.05, ***p<0.001. Statistical analysis was performed by one-way ANOVA for untreated and treated samples. **(a)** Normal brain vs Vehicle: p=0.0472, Vehicle vs DFMO: p<0.0001, Vehicle vs DFMO/AMXT 1501: p<0.0001, AMXT 1501 vs DFMO/AMXT 1501: p<0.0001. **(b)** Normal brain vs Vehicle: p=0.0457 Vehicle vs DFMO/AMXT 1501: p=0.0332.

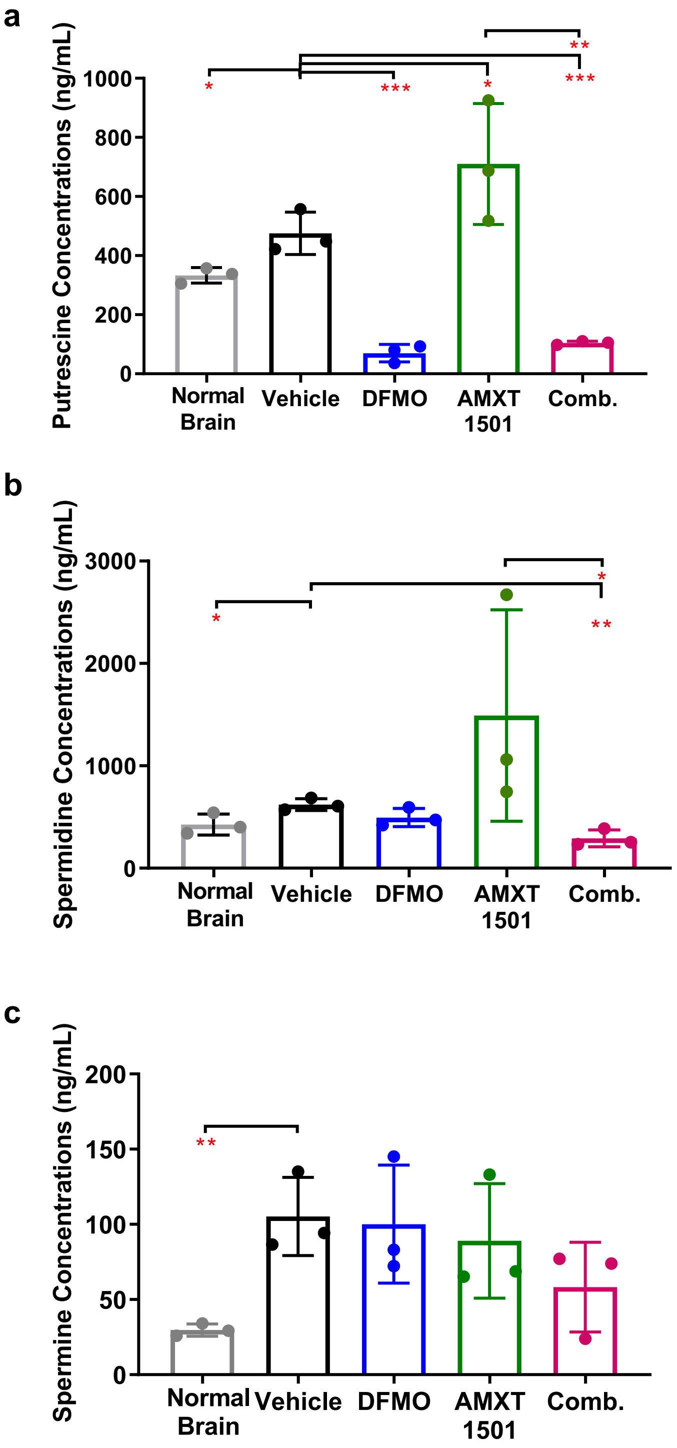

Supplementary Figure 25: Plasma polyamine concentrations of Balb/C Nude mice. (a) Putrescine, **(b)** spermidine and **(c)** spermine levels were significantly increased in the plasma of mice with tumors, compared to mice without tumors. The combination treatment of DFMO and AMXT 1501 potently decreased polyamine concentrations compared to untreated and single agents. Samples were collected from three mice from each cohort. Data is presented as means ± SEM. *p<0.05, **p<0.01, ***p<0.001 Statistical analysis was performed by one-way ANOVA for untreated and treated samples. **(a)** Normal brain vs Vehicle: p=0.0320, Vehicle vs DFMO: p=0.008, Vehicle vs AMXT 1501: p=0.0343, Vehicle vs DFMO/AMXT 1501: p=0.0009, AMXT 1501 vs DFMO/AMXT 1501: p=0.0068. **(b)** Normal brain vs Vehicle: p=0.0463, Vehicle vs DFMO/AMXT 1501: p=0.0048, AMXT 1501 vs DFMO/AMXT 1501: p=0.0439. **(c)** Normal brain vs Vehicle: p=0.0076.

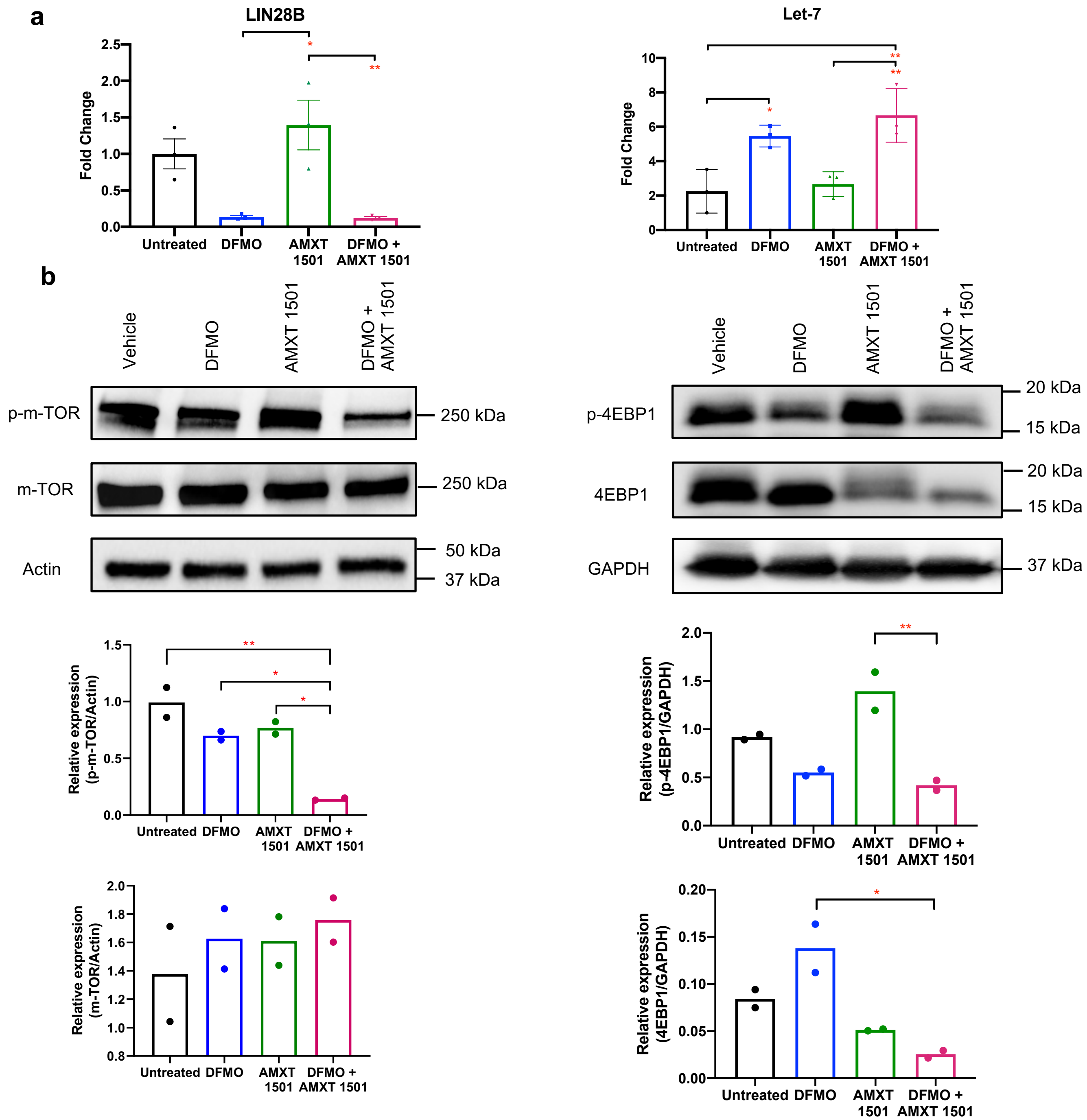

Supplementary Figure 26: Combination treatment influences protein translation through integration of Lin28B/let7 axis and mTORC1. In HSJD-DIPG007 cells: **(a)** DFMO/AMXT 1501 combination treatment decreased LIN28B and increased let-7 mRNA levels. Data is presented as means ± SEM of three independent experiments. **(b)** Treatment with DFMO and AMXT 1501 resulted in decreased p-m-TOR protein expression. Polyamine transport inhibition via AMXT 1501 led to an increase in ODC1 expression. The combination treatment resulted in decreased ODC1 expression. Densitometric analysis of protein bands from 2 independent experiments. *p<0.05, **p<0.01. Statistical analysis was performed by one-way ANOVA for untreated and treated samples. **(a)** LIN28B: DFMO vs AMXT 1501: p=0.0040, AMXT 1501 vs DFMO/AMXT 1501: p=0.0086. Let-7: Untreated vs DFMO: p=0.0406, Untreated vs DFMO/AMXT 1501: p=0.0096, AMXT 1501 vs DFMO/AMXT 1501: p=0.0154. **(b)** p-MTOR: Untreated vs DFMO/AMXT 1501: p=0.0043, DFMO vs DFMO/AMXT 1501: p=0.0200, AMXT 1501 vs DFMO/AMXT 1501: p=0.0133. p-4EBP1: AMXT 1501 vs DFMO/AMXT 1501: =0.0094. 4EBP1: DFMO vs DFMO/AMXT 1501: p=0.0157.

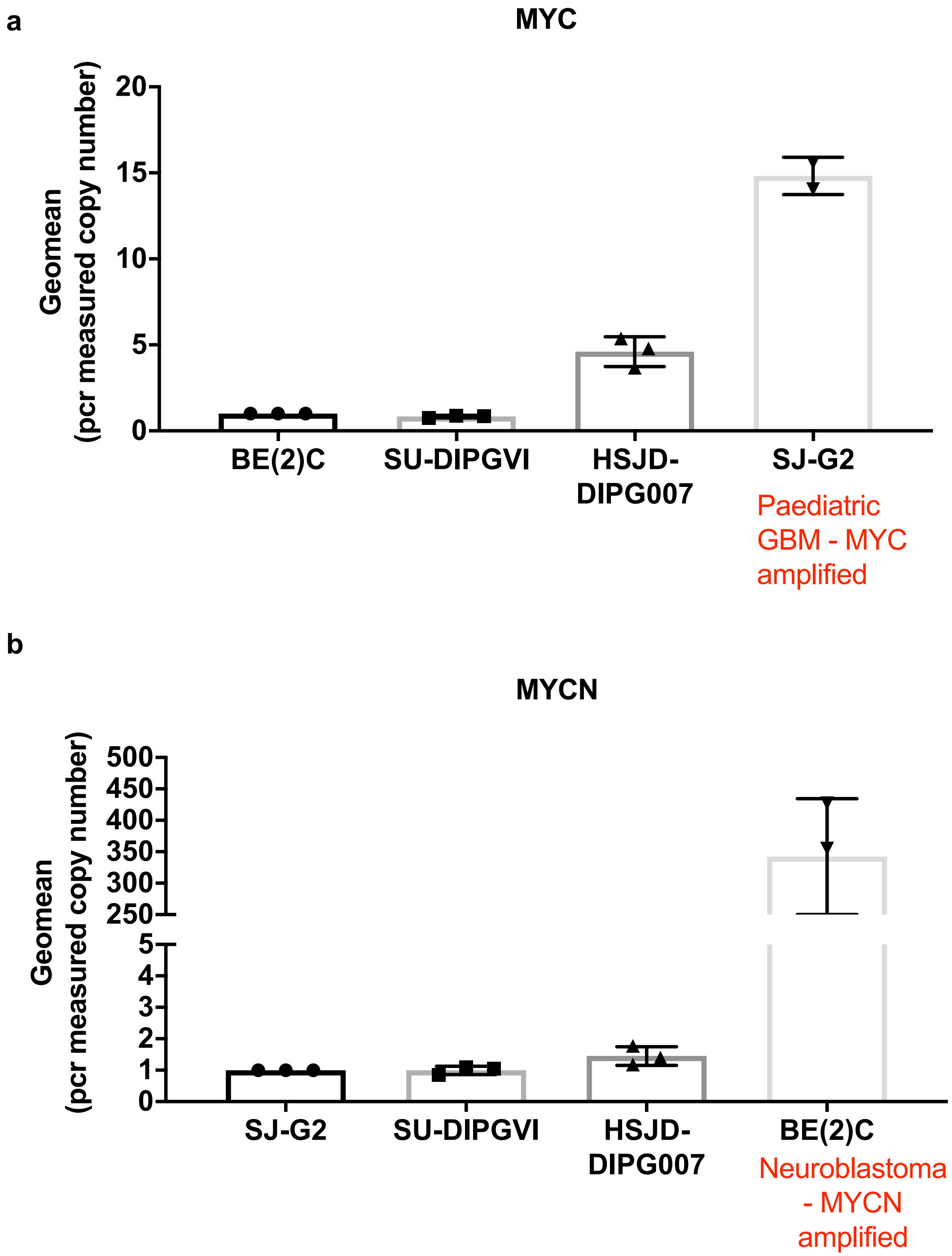

Supplementary Figure 27: MYC/MYCN copy number in DIPG cell lines. **(a)** SU-DIPGVI has a single copy of MYC. HSJD-DIPG007 has approximately >4 copies of MYC gene. SJ-G2 was used as positive control, being an established MYC-amplified cell line, and BE(2)C was used as a negative control. **(b)** SU-DIPGVI and HSJD-DIPG007 have a single copy of MYCN. BE(2)C was used as positive control, being an established MYCN-amplified cell line, and SJ-G2 was used as a negative control. Data is presented as means ± SD of three independent experiments.

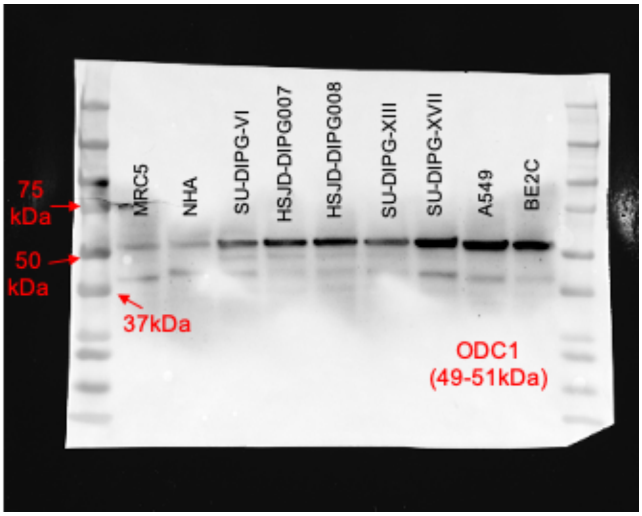

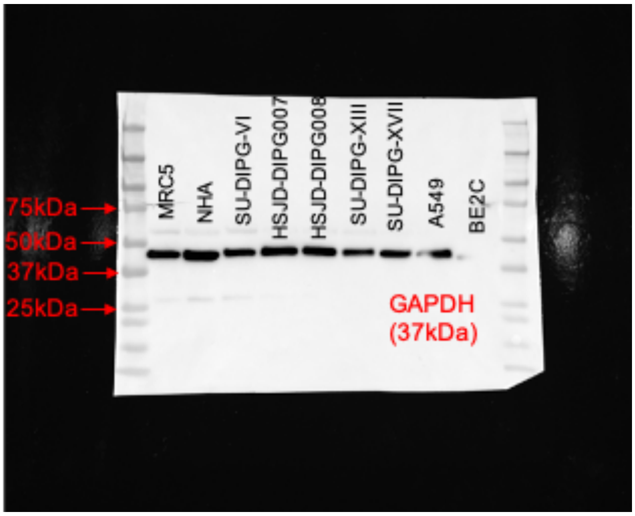

Supplementary Figure 28: Full scan representative images of Figure 1c for ODC1 and GAPDH.

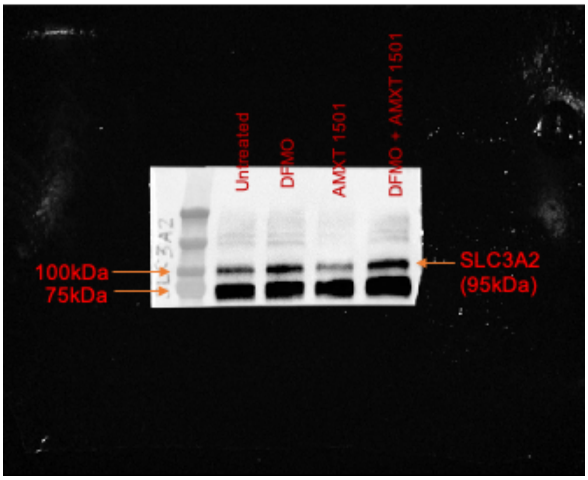

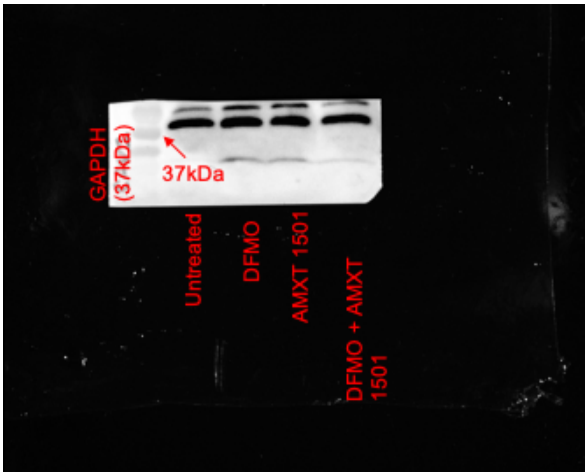

Supplementary Figure 29: Full scan representative images of Figure 2d for SLC3A2 and GAPDH.

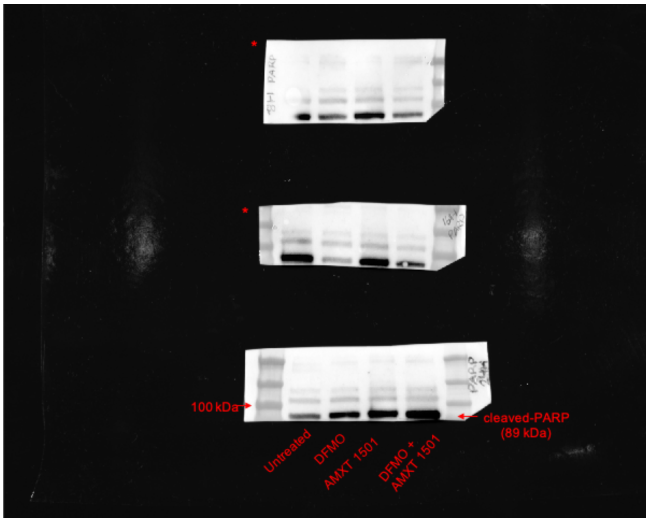

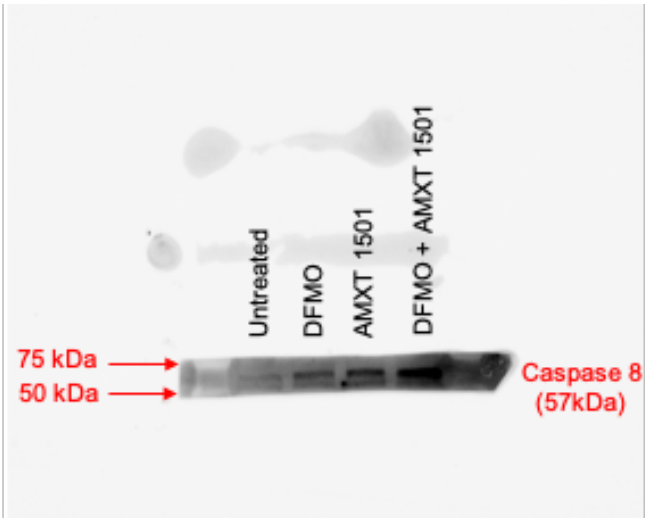

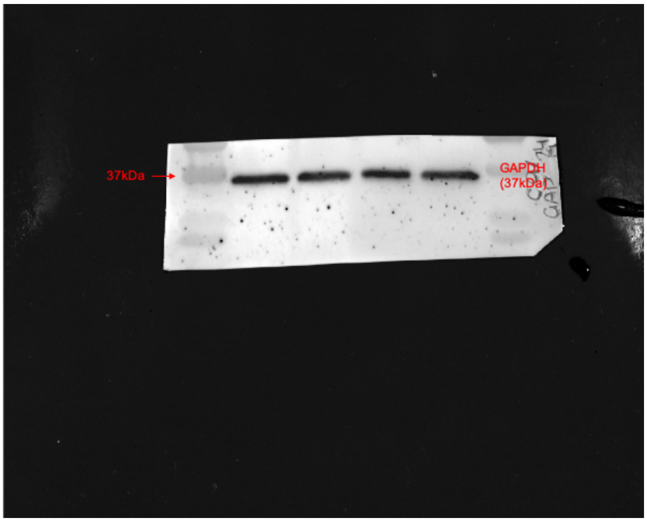

Supplementary Figure 30: Full scan representative images of Figure 4e for cleaved-PARP, caspase 8 and GAPDH. *These membranes were imaged at the same time but are not related to the prepared figure.

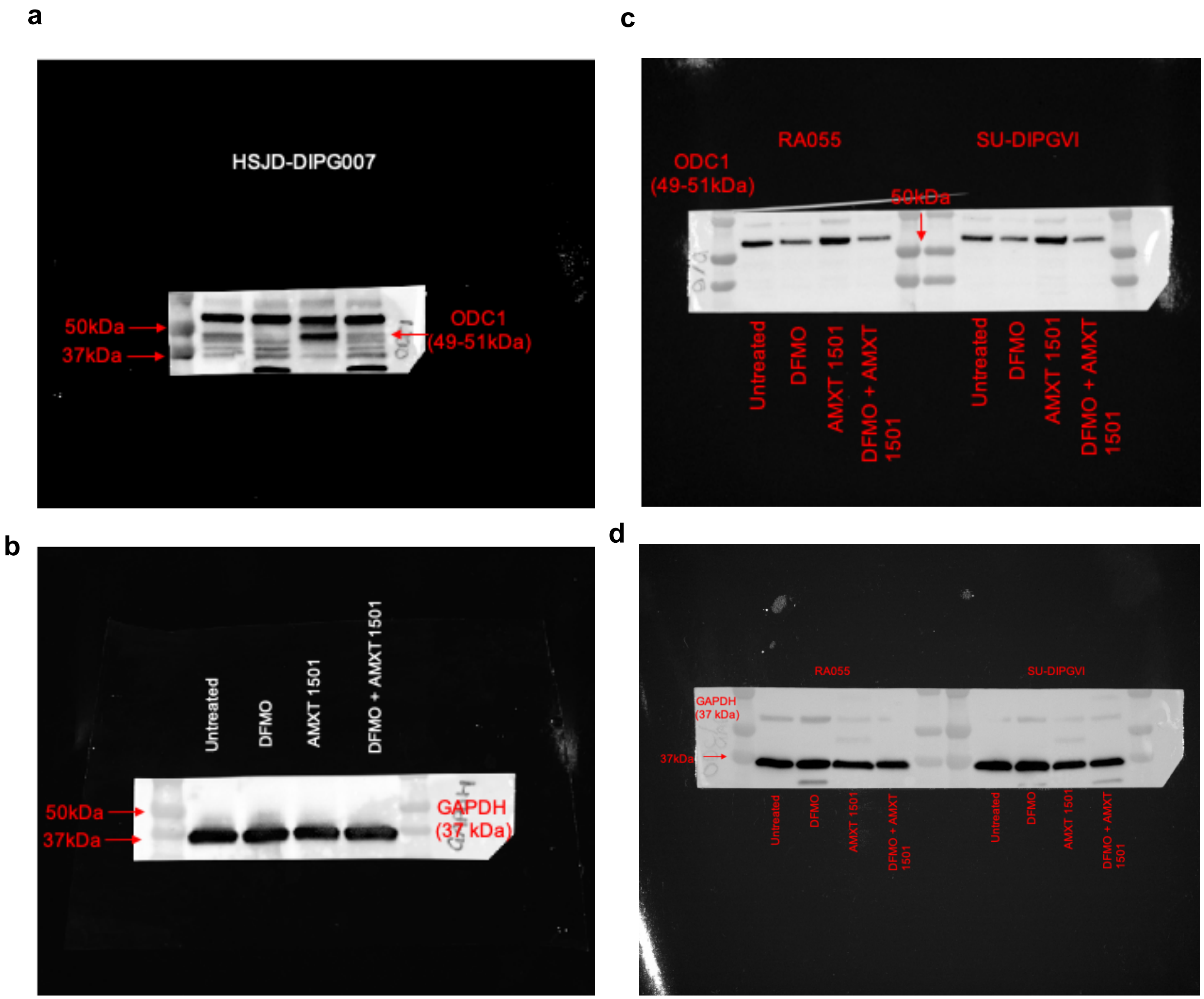

Supplementary Figure 31: Full scan representative images of Supplementary Figure 11. HSJD DIPG007: **(a)** ODC1 and **(b)** GAPDH. RA055 and SU-DIPGVI: **(c)** ODC1 and **(d)** GAPDH.

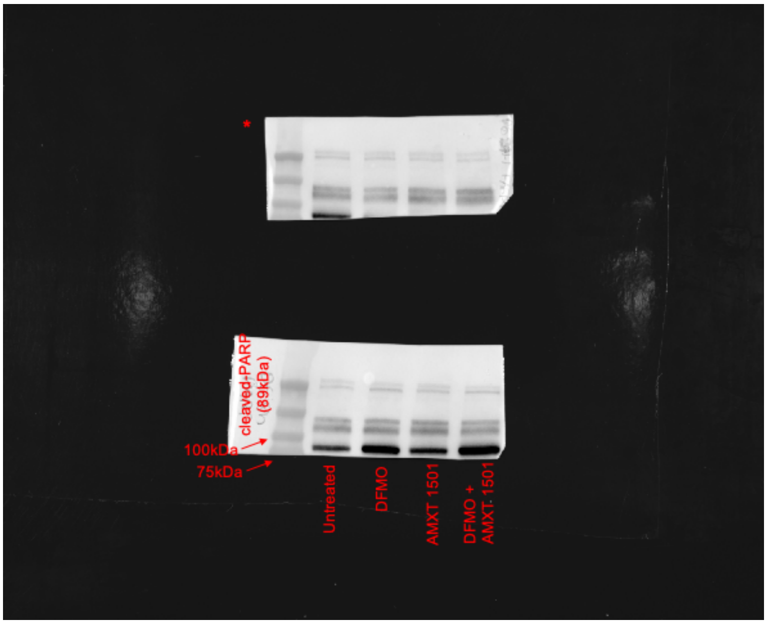

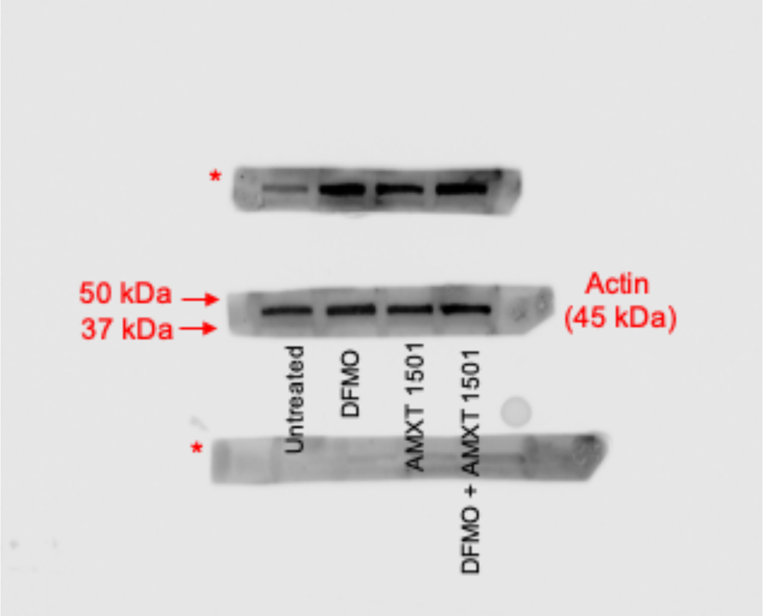

Supplementary Figure 32: Full scan representative images of Supplementary Figure 16b for cleaved-PARP and Actin. *These membranes were imaged at the same time but are not related to the prepared figure.

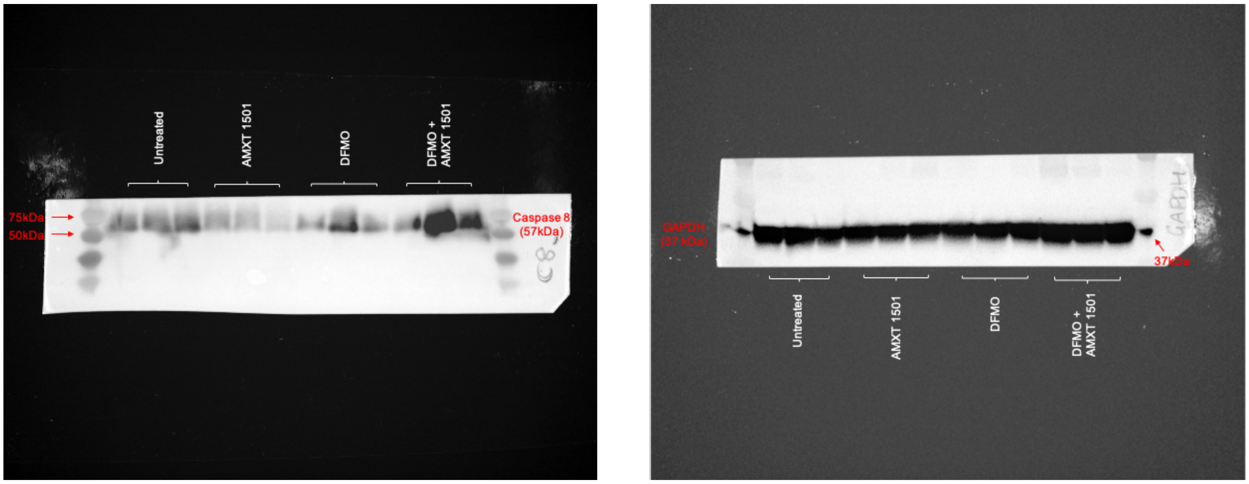

Supplementary Figure 33: Full scan representative images of Supplementary Figure 19 for caspase 8 and GAPDH.

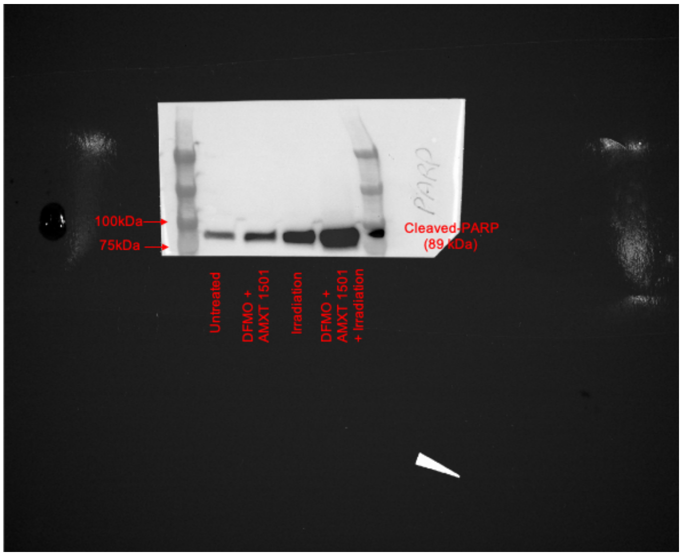

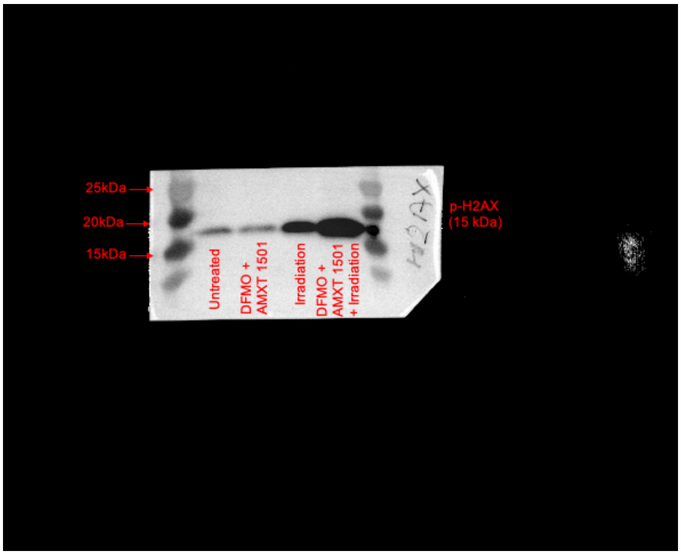

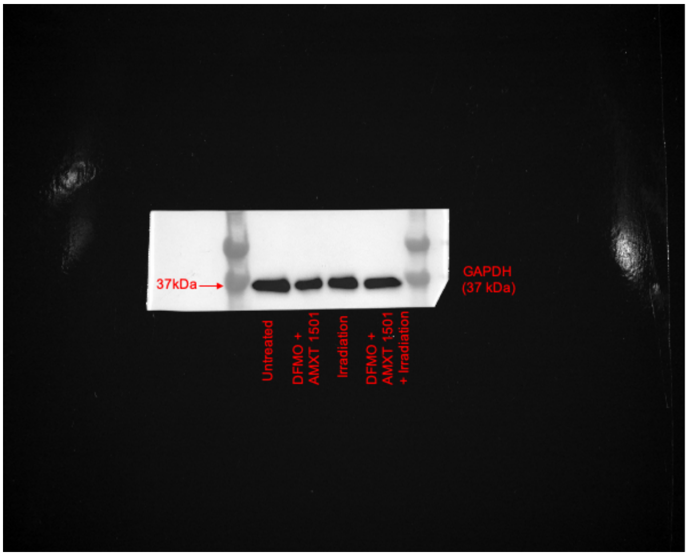

Supplementary Figure 34: Full scan representative images of Supplementary Figure 20c for cleaved-PARP, p-H2AX and GAPDH.

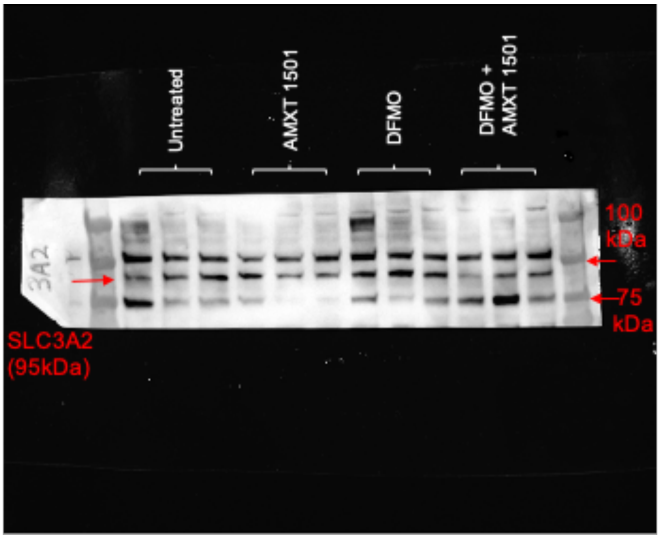

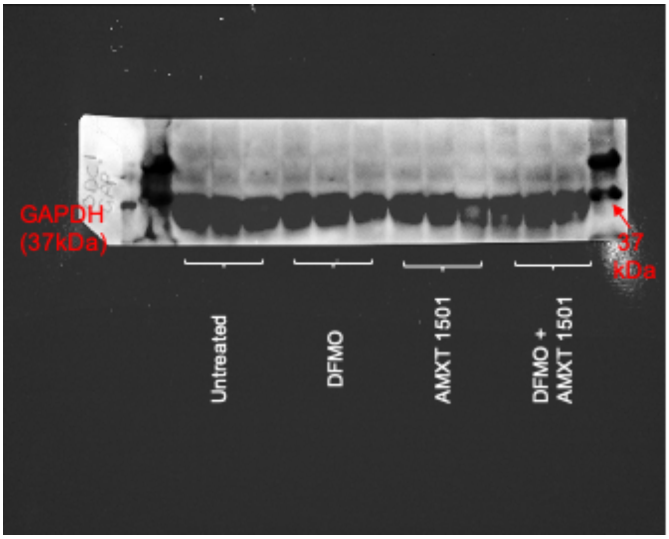

Supplementary Figure 35: Full scan representative images of Supplementary Figure 23 for SLC3A2 and GAPDH.

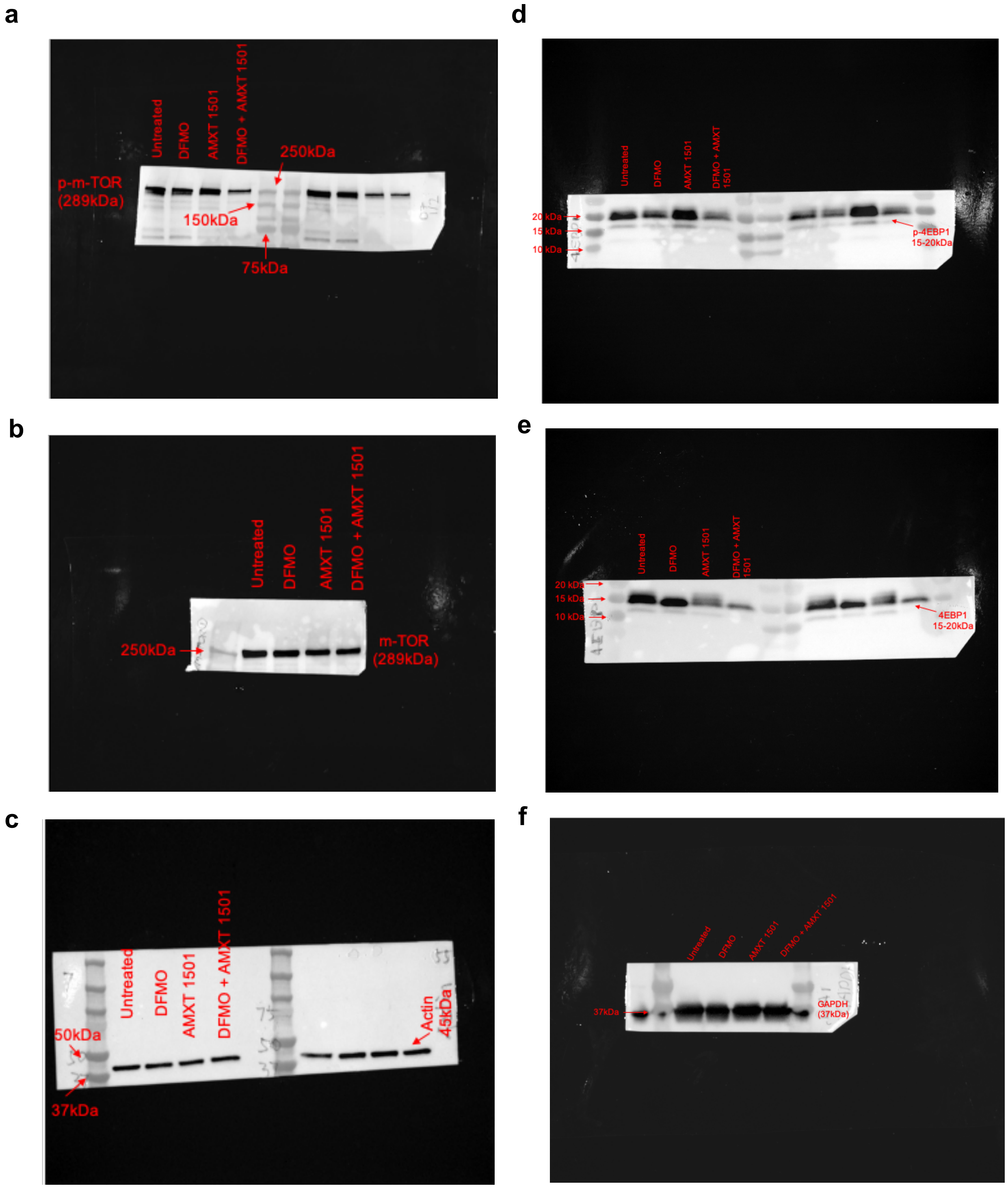

Supplementary Figure 36: Full scan representative images of Supplementary Figure 26b for **(a)** p-mTOR, **(b)** m-TOR, **(c)** Actin, and **(d)** p-4EBP1, **(e)** 4EBP1, **(f)** GAPDH.

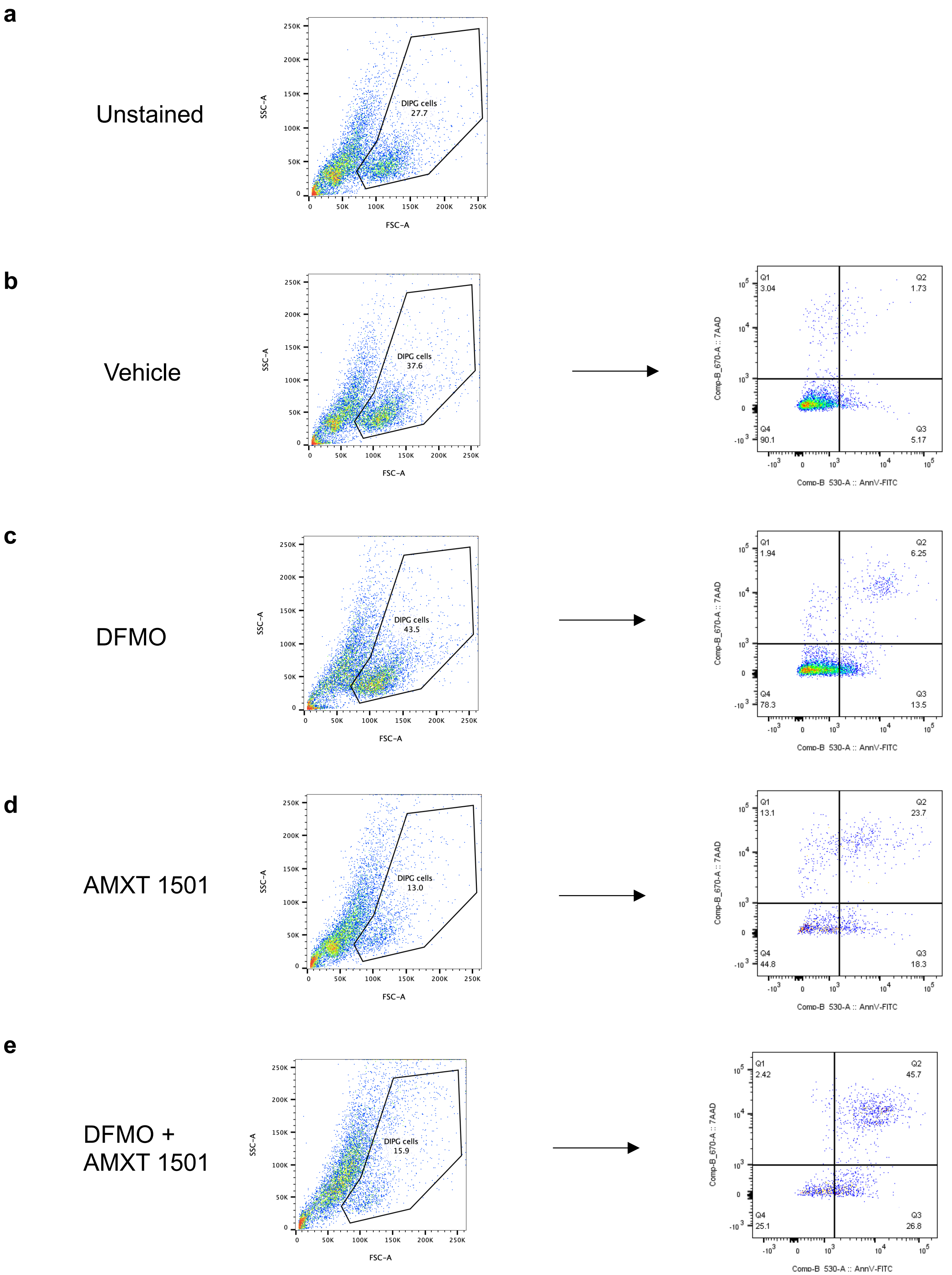

Supplementary Figure 36: Flow cytometry gating strategy utilised to identify apoptotic cells. After 24h treatment, DIPG cells were resuspended in Annexin binding buffer containing Annexin V-FITC and 7AAD and FACS analysis was conducted on Facs Canto where 10,000 events were collected for each sample. Unstained cells were also run through flow as a control. Gating strategy to determine to the percentage of apoptotic/late apoptotic DIPG cells in in **(a)** unstained, **(b)** Vehicle, **(c)** DFMO, **(d)** AMXT 1501 and **(e)** DFMO + AMXT 1501 cohorts.

Supplementary Table 1: Expression values for biosynthetic polyamine pathway genes in Normal Brain (n=11) and DIPG/DMG (n=49) samples.

ODC1		SRM		SMS		AZIN1		AMD1		SMOX	
Normal Brain	DIPG/ DMG	Normal Brain	DIPG/ DMG	Normal Brain	DIPG/ DMG	Normal Brain	DIPG/ DMG	Normal Brain	DIPG/ DMG	Normal Brain	DIPG/ DMG
4.293	6.663	3.993	7.049	3.708	5.988	5.033	7.198	3.823	5.941	2.109	6.372
4.471	5.177	4.084	6.780	3.712	5.202	4.660	5.841	3.525	4.324	1.994	5.425
3.901	4.776	4.401	6.548	4.415	7.416	5.033	7.152	3.403	6.417	2.256	5.644
4.018	5.271	4.148	6.872	4.223	6.938	3.246	5.634	3.394	5.836	2.523	5.853
4.242	4.592	4.223	6.970	4.243	5.119	4.972	7.102	3.625	5.416	2.470	6.727
4.283	5.981	4.127	7.541	3.864	6.350	4.319	7.713	3.227	6.372	2.488	5.685
3.969	5.877	4.375	6.355	4.827	5.467	3.741	6.955	3.083	5.412	2.474	7.051
3.729	4.833	4.722	6.544	4.275	6.401	4.940	7.009	3.177	5.291	2.200	7.228
4.435	2.942	3.888	6.261	3.636	7.019	4.302	6.826	3.127	5.309	2.177	6.577
4.324	6.331	4.611	6.409	3.382	6.820	5.059	7.324	3.347	5.950	1.849	5.881
4.083	6.744	4.181	6.313	3.797	5.646	4.533	6.601	3.509	5.051	2.525	6.651
	7.443		7.025		5.853		6.991		5.741		6.396
	6.046		7.044		5.881		6.910		6.199		5.631
	4.767		6.417		6.279		6.384		4.725		6.565
	5.261		7.084		5.736		6.073		5.303		5.648
	6.556		6.947		6.809		6.992		5.593		5.951
	6.719		6.719		6.356		8.067		5.833		6.862
	7.683		5.922		8.192		5.622		6.058		6.365
	5.937		7.049		5.545		7.033		5.805		6.313
	8.891		7.379		7.028		7.536		5.195		7.036
	3.785		6.457		5.763		6.468		4.532		7.368
	8.148		6.141		6.029		6.492		6.016		7.328
	6.871		6.813		7.068		7.378		5.614		7.406
	8.436		5.567		7.552		6.810		5.691		6.989
	6.530		6.236		6.024		6.448		5.274		6.941
	7.540		6.661		5.937		6.561		5.086		5.718
	7.964		7.292		6.156		7.089		6.186		6.204
	8.181		6.932		6.747		7.661		5.512		5.216
	6.825		2.808		4.736		5.822		5.042		4.874
	7.224		3.149		4.819		5.309		4.983		2.269
	9.515		3.638		4.156		5.388		5.014		3.238
	7.938		3.380		4.877		4.973		4.121		3.117
	7.833		2.455		4.839		6.111		4.030		3.044
	7.709		4.015		4.029		5.736		4.810		3.514
	7.713		3.665		4.574		5.508		4.416		3.792
	8.396		3.830		4.345		5.714		5.374		4.822
	8.530		2.839		4.523		2.853		1.388		2.485
	9.558		3.903		4.156		5.619		4.320		3.952
	6.651		4.084		3.992		5.885		5.424		2.198
	8.438		5.296		6.463		6.055		5.928		2.416
	8.845		2.831		3.686		5.290		4.471		3.704
	6.652		3.692		5.118		4.646		4.008		3.302
	6.389		3.639		4.172		5.332		3.716		3.545
	8.479		4.278		5.668		5.953		5.914		2.234
	7.343		4.661		5.627		6.255		5.792		2.415
	6.612		7.017		7.864		6.018		6.149		2.259
	7.285		3.207		7.203		5.686		5.259		2.277
	8.062		5.251		5.522		6.018		5.826		1.812
	7.806		3.186		3.974		4.935		4.816		2.706

Supplementary Table 2: Expression values for catabolic polyamine pathway genes in Normal Brain (n=11) and DIPG/DMG (n=49) samples.

SAT1		PAOX		OAZ1		OAZ2		OAZ3	
Normal Brain	DIPG/ DMG	Normal Brain	DIPG/ DMG	Normal Brain	DIPG/ DMG	Normal Brain	DIPG/ DMG	Normal Brain	DIPG/ DMG
7.981	7.183	3.447	2.198	9.404	5.661	7.092	7.118	0.759	0.885
7.007	6.727	3.154	2.018	9.703	5.500	7.109	7.603	1.390	1.017
5.997	5.740	3.626	0.566	9.542	5.493	7.487	7.073	1.517	1.140
5.622	5.373	3.257	1.811	9.325	5.840	7.119	7.409	0.999	0.657
6.360	5.765	2.558	2.740	9.010	5.740	6.487	3.692	1.563	0.493
6.020	5.614	3.199	1.872	9.557	6.102	7.714	3.909	1.382	1.053
5.910	6.406	2.688	1.761	5.781	6.486	3.457	3.879	1.267	1.838
5.422	6.815	3.165	2.124	6.449	3.934	3.228	3.527	1.912	0.949
7.428	4.628	3.298	1.718	6.262	5.458	3.430	4.019	0.798	0.043
5.346	5.927	2.720	1.561	6.611	6.444	2.804	3.987	1.324	1.088
5.894	5.285	2.541	3.021	6.132	6.699	3.148	4.132	1.497	1.186
	4.606		2.698		5.097		4.214		2.029
	4.853		2.141		6.249		3.956		0.553
	7.981		2.362		6.010		3.610		0.395
	7.209		2.390		6.027		4.038		1.371
	4.489		1.949		5.625		3.180		1.996
	4.757		1.960		6.395		3.613		1.606
	5.086		1.104		7.539		4.000		3.232
	4.420		3.044		6.713		1.970		2.445
	7.271		1.454		6.546		3.167		3.283
	4.670		2.452		6.646		3.325		2.110
	2.130		4.087		6.601		3.140		*
	1.971		2.546		6.732		1.941		
	1.867		1.465		7.085		2.267		
	2.145		2.744		6.771		2.950		
	1.837		3.036		6.100		3.266		
	1.837		3.064		4.935		2.756		
	1.937		2.539		5.853		3.978		
	2.168		-0.067		6.912		3.553		
	2.292		0.374		6.308		3.349		
	2.193		1.823		4.717		3.858		
	1.920		0.075		6.096		3.972		
	1.924		-0.289		5.018		3.904		
	1.914		1.564		5.630		3.525		
	2.217		1.969		7.099		3.728		
	1.784		2.641		5.751		3.694		
	1.958		1.222		6.356		3.968		
	1.848		1.733		4.658		3.769		
	2.386		-1.135		6.355		3.898		
	1.732		1.003		5.330		3.685		
	2.133		-0.286		5.035		3.880		
	2.053		-0.969		5.735		3.805		
	1.896		2.131		6.491		3.482		
	2.352		2.014		6.569		3.709		
	2.622		1.087		6.574		3.884		
	2.014		-2.957		5.551		3.541		
	2.179		1.398		5.340		3.737		
	1.890		0.004		4.563		3.262		
	2.162		2.321		4.646		3.497		

*no values were available from ZCC platform for OAZ3 gene.

Supplementary Table 3: Expression values for catabolic polyamine pathway genes in Normal Brain (n=11) and DIPG/DMG (n=49) samples.

SLC3A2					
Normal Brain (n=11)	DIPG/DMG (n=49)				
3.869	6.195	4.232	6.175	7.343	8.454
4.206	6.200	4.627	7.314	7.891	6.631
4.073	5.180	4.860	7.002	6.599	6.619
3.848	6.326	5.265	7.428	7.959	7.167
4.007	6.154	5.257	6.733	7.429	7.782
3.868	4.609	3.468	7.792	7.569	
3.893	4.848	6.104	8.203	7.441	
4.247	5.499	4.274	8.355	8.548	
4.041	2.915	4.515	8.139	7.628	
3.868	4.672	5.005	7.384	7.516	
4.144	4.334	7.276	7.096	7.990	

Supplementary Table 4: Expression values for SLC3A2 transporter gene from ZCC platform.

SLC3A2									
DMG (n=28)	Other Paediatric Cancers (n=148)					HGG (n=32)	High-risk Neuroblastoma (n=17)	Other Brain Tumours (n=34)	
155.02	181.95	96.29	71.24	67.05	49.8	205.38	75.49	371.19	122.91
72.27	63.12	171.04	65.12	96.25	180.4	290.82	105.89	80.71	200.77
159.16	79.2	47.92	110.42	75.82	128.47	174.56	147.95	156.83	387.63
128.18	126.54	133.17	81.51	48.21	50.16	197.46	71.8	118.59	126.44
172.21	113.76	199.11	209.52	62.06	149.46	242.79	83.74	187.45	168.4
106.37	120.27	79.89	100.24	41.21	61.13	284.2	57.88	91.76	96.61
221.56	149.23	86.53	47.73	42.24	118.53	81.8	71.24	87.74	83.42
294.69	79.86	86.91	76.38	76.48	137.97	269.58	65.12	483.7	74.93
327.35	182.42	183.37	113.88	96.55	90.28	250.13	110.42	232.35	42.44
281.84	159.34	101.4	83.64	236.59	116.47	135.01	81.51	237.03	81.65
167.03	66.01	203.18	114.99	51.63	119	246.06	209.52	156.68	63.41
136.81	76.5	75.78	228.54	100.74	170.57	171.09	100.24	72.79	67.62
162.34	61.4	79.34	127.56	28.82	486.65	74.29	47.73	191.44	48.82
237.3	51.32	30.14	149.28	118.92	25.78	258.11	76.38	104.47	75.92
96.94	50.86	82.46	120.16	179.27	108.28	173.33	113.88	107.79	180.39
248.88	108.31	83.35	162.37	71.53	199.99	299.79	83.64	262.86	138.45
172.33	107.05	52.94	151.42	170.42	93.77	120.38	88.26	183.28	128.87
189.83	93.78	101.71	76.39	196.42	58.2	203.58			
173.74	78.33	298.72	50.92	134.49	95.14	269.74			
374.31	145.66	135.39	331.98	95.76	64.73	203.7			
197.84	67.94	97.73	139.89	59.54	108.19	240.09			
182.99	93.73	183.66	84.21	154.94	136.97	217.61			
254.23	165.86	76.2	103.9	91.04	236.31	228.98			
350.69	53.47	88.26	91.38	85.03	110.67	178.92			
99.11	60.76	75.49	78.79	77.9	71.04	145.72			
98.32	94.88	105.89	58.18	53.01	96.88	137.36			
143.72	50.47	147.95	34.28	59.61	69.72	183.82			
220.15	229.99	71.8	41.05	245.73	70.5	267.74			
	97.17	83.74	51.02	67.3		242.17			
	160.53	57.88	88.71	159.92		264.17			
						335.8			
						259.53			

Supplementary Table 5: Key characteristics of primary brain tumour cultures used for in vitro and in vivo studies.

Name	Tumour type	Autopsy/ Biopsy	Treatment	Key Molecular Characteristics
**SU-DIPGVI**[$]	DIPG	Autopsy	IR, vorinostat	H3.3K27M, TP53 mutation
**HSJD-DIPG007**[*]	DIPG	Autopsy	Not specified	H3.3K27M, ACVR1-R206H, PIK3CA mutation
**HSJD-DIPG008**[*]	DIPG	Autopsy	Not specified	H3.3-K27M
**VUMC-DIPG10**[$]	DIPG	Autopsy	IR, gemcitabine	H3wt, NF1 mutation, MYCN amplification
**HSJD-DIPG011**[#]	DIPG	Autopsy	unknown	H3.3K27M
**HSJD-DIPG012**[#]	DIPG	Autopsy	unknown	H3.3K27M
**HSJD-DIPG013**[#]	DIPG	Autopsy	unknown	H3.3K27M
**SU-DIPGXVII**[$]	DIPG	Autopsy	IR, avastin, panobinostat, temsirolimus	H3.3-K27M, TP53 mutation
**RA055**[&]	DIPG	Biopsy	No	H3.3K27M, TP53 mutation, PDGFRa amplification, MYCN amplification
**AUS-DIPG017**[&]	Thalamic astrocytoma	Autopsy	IR, temozolomide	H3wt, TP53 mutation, CDKN2A/B loss
**P001805**[&]	Thalamic DMG	Biopsy	No	H3.3K27M, TP53 mutation

[$]Nagaraja S, et al, Transcriptional dependencies in Diffuse Intrinsic Pontine Glioma. 2017, Cancer Cell, 31(5): 635-652.

*Mackay A, et al, Integrated Molecular Meta-Analysis of 1,000 Pediatric High-Grade and Diffuse Intrinsic Pontine Glioma. 2017, Cancer Cell, 2(4):520-537

[#]Meel MH, et al, Culture methods of diffuse intrinsic pontine glioma cells determine to targeted therapies. Experimental Cell Research, 2017, 60(2): 397-403

Benitez-Ribas D, et al, Immune Response Generated With the Administration of Autologous Dendritic Cells Pulsed With an Allogenic Tumoral Cell-Lines Lysate in Patients With Newly Diagnosed Diffuse Intrinsic Pontine Glioma Frontiers in Oncology, 8: 127.

Qi J, et al, Tenascin-C expression contributes to pediatric brainstem glioma tumor phenotype and represents a novel biomarker of disease, 2019, Acta Neuropathologica Communications, 2019, 7: 75.

[&]Zero Childhood Cancer Precision Medicine Platform & HOTRODS Biological Study of Diffuse Intrinsic Pontine Gliomas (DIPG) and Early Phase Trial

Supplementary Table 6: Biochemical analysis performed in blood samples from Balbc/Nude animals treated with 3 different doses of AMXT 1501. Normal reference ranges were obtained from Charle's River Laboratory for 8-10 weeks old non-fasted female mice. * Biochemical assessment measurement is different from normal ranges or vehicle values.

Marker	Normal Ranges	Vehicle	AMXT 1501	DFMO+ AMXT 1501	AMXT 1501	DFMO+ AMXT 1501	AMXT 1501
			(dicaprate) 5mg/kg	(dicaprate) 5mg/kg	(dicaprate) 7.5mg/kg	(dicaprate) 7.5mg/kg	(dicaprate) 10mg/kg
ALB (g/L)	32-43	43	40	37	40	37	41
ALP (U/L)	63-178	110	78	56	49*	63	16*
ALT (U/L)	39-90	45	45	38	53	59	49
TBIL (umol/L)	3.4-10.3	4	5	5	4	5	HEM
BUN (mmol/L)	6.1-13.6	5.2	7.2	8	7.6	8.1	8.9
CA (mmol/L)	2.4-2.9	2.45	2.49	2.6	2.49	2.4	2.64
PHOS (mmol/L)	2.3-4.3	2.11	1.76	1.621	1.7	1.53	1.92
CRE (umol/L)	26.5-44.2	<18	<18	<18	<18	<18	27
GLU (mmol/L)	7.4-13.4	6	2.9*	4.6*	3.5*	3.6*	2.3 *
NA+ (mmol/L)	145.3-173.6	148	147	150	146	149	153
K+ (mmol/L)	8.1-14.4	HEM	>8.5	7.3	>8.5	>8.5	HEM
TP (g/L)	53-68	55	58	54	56	56	62

ALB: albumin, ALP: alkaline phosphatase, ALT: alanine aminotransferase, TBIL: total bilrubin, BUN: urea nitrogen, CA: total calcium, PHOS: phosphorus, CRE: creatinine, GLU: glucose, TP: total protein, HEM: hemolysis

Supplementary Table 7: Multiple comparisons test for SU-DIPGVI-LUC orthotopic animal model treated with polyamine pathway inhibitors.

Comparison	p-value from log-rank test	q-value from 'compare a stack of p-values'	Discovery?
Vehicle vs DFMO	0.0041	0.0010	Yes
Vehicle vs AMXT 1501	0.3263	0.0659	No
Vehicle vs DFMO/AMXT 1501	<0.0001	3.3667e-005	Yes
DFMO vs DFMO/AMXT 1501	<0.0001	3.3667e-005	Yes
AMXT 1501 vs DFMO/AMXT 1501	<0.0001	3.3667e-005	Yes

Supplementary Table 8: Multiple comparisons test for HSJD-DIPG007 orthotopic animal model treated with polyamine pathway inhibitors.

Comparison	p-value from log-rank test	q-value from 'compare a stack of p-values'	Discovery?
Vehicle vs DFMO	0.0001	2.5250e-005	Yes
Vehicle vs AMXT 1501	0.0884	0.0179	No
Vehicle vs DFMO/AMXT 1501	<0.0001	2.5250e-005	Yes
DFMO vs DFMO/AMXT 1501	<0.0001	2.5250e-005	Yes
AMXT 1501 vs DFMO/AMXT 1501	<0.0001	2.5250e-005	Yes

Supplementary Table 9: Multiple comparisons test for RA055 orthotopic animal model treated with polyamine pathway inhibitors.

Comparison	p-value from log-rank test	q-value from 'compare a stack of p-values'	Discovery?
Vehicle vs DFMO	0.0003	0.0001	Yes
Vehicle vs AMXT 1501	0.0708	0.0179	No
Vehicle vs Irradiation	0.0001	3.3667e-005	Yes
Vehicle vs DFMO/AMXT 1501	0.0001	3.3667e-005	Yes
DFMO vs DFMO/AMXT 1501	0.0001	3.3667e-005	Yes
AMXT 1501 vs DFMO/AMXT 1501	0.0001	3.3667e-005	Yes
DFMO/Irradiation vs DFMO/AMXT 1501/Irradiation	0.0001	3.3667e-005	Yes
AMXT 1501/Irradiation vs DFMO/AMXT 1501/Irradiation	0.0001	3.3667e-005	Yes
DFMO/AMXT 1501 vs DFMO/AMXT 1501/Irradiation	0.4557	0.1023	No
