# Supplementary material for: Dual targeting of polyamine synthesis and uptake in diffuse intrinsic pontine gliomas
Source: Nat Commun. 2021 Feb 12;12:971. doi: 10.1038/s41467-021-20896-z (PMC7881014; doi:10.1038/s41467-021-20896-z)
Supplement: Supplementary file 3 — Reporting Summary [file 41467_2021_20896_MOESM3_ESM.pdf]

nature research

Corresponding author(s): David S. Ziegler

Last updated by author(s): Dec 18, 2020

Reporting Summary

Nature Research wishes to improve the reproducibility of the work that we publish. This form provides structure for consistency and transparency in reporting. For further information on Nature Research policies, see Authors & Referees and the Editorial Policy Checklist .

Statistics

For all statistical analyses, confirm that the following items are present in the figure legend, table legend, main text, or Methods section.

n/a	Confirmed	
☐	☒	The exact sample size (*n*) for each experimental group/condition, given as a discrete number and unit of measurement
☐	☒	A statement on whether measurements were taken from distinct samples or whether the same sample was measured repeatedly
☐	☒	The statistical test(s) used AND whether they are one- or two-sided<br>*Only common tests should be described solely by name; describe more complex techniques in the Methods section.*
☒	☐	A description of all covariates tested
☐	☒	A description of any assumptions or corrections, such as tests of normality and adjustment for multiple comparisons
☐	☒	A full description of the statistical parameters including central tendency (e.g. means) or other basic estimates (e.g. regression coefficient) AND variation (e.g. standard deviation) or associated estimates of uncertainty (e.g. confidence intervals)
☐	☒	For null hypothesis testing, the test statistic (e.g. *F*, *t*, *r*) with confidence intervals, effect sizes, degrees of freedom and *P* value noted<br>*Give P values as exact values whenever suitable.*
☒	☐	For Bayesian analysis, information on the choice of priors and Markov chain Monte Carlo settings
☒	☐	For hierarchical and complex designs, identification of the appropriate level for tests and full reporting of outcomes
☐	☒	Estimates of effect sizes (e.g. Cohen's *d*, Pearson's *r*), indicating how they were calculated

Our web collection on statistics for biologists contains articles on many of the points above.

Software and code

Policy information about availability of computer code

Data collection	No software was used.
Data analysis	Prism v8, FlowJo, Calcusyn v2.0 , Image J v1.52

For manuscripts utilizing custom algorithms or software that are central to the research but not yet described in published literature, software must be made available to editors/reviewers. We strongly encourage code deposition in a community repository (e.g. GitHub). See the Nature Research guidelines for submitting code & software for further information.

Data

Policy information about availability of data

All manuscripts must include a data availability statement. This statement should provide the following information, where applicable:

- Accession codes, unique identifiers, or web links for publicly available datasets
- A list of figures that have associated raw data
- A description of any restrictions on data availability

The ZCC RNA-seq data are available in the European Genome-phenome Archive (EGA) database under the accession code EGAS00001004905 [https://ega-archive.org/studies/EGAS00001004905]. These data are available under controlled access. In order to gain access to the data, a request must be made to the Data Access Committee at EGA who will forward the appropriate approval documents that will be reviewed by the ZERO childhood cancer Research Management Committee. All the other data supporting the findings of this study are available within the article and its supplementary information files and from the corresponding author upon reasonable request. A reporting summary for this article is available as a Supplementary Information file.

Field-specific reporting

Please select the one below that is the best fit for your research. If you are not sure, read the appropriate sections before making your selection.

☒ Life sciences ☐ Behavioural & social sciences ☐ Ecological, evolutionary & environmental sciences

For a reference copy of the document with all sections, see nature.com/documents/nr-reporting-summary-flat.pdf

Life sciences study design

All studies must disclose on these points even when the disclosure is negative.

Sample size	We determined sample size based on power calculations. For the F test with assumption of alpha 0.05, power=0.80 and effective size 0.50, the minimum number of animals in each cohort is 12 mice per group.
Data exclusions	No in vitro data was excluded, and only two mice were excluded from the three in vivo studies (RA055 xenograft). These mice were excluded due to development of a rash prior to tumor engraftment. Our ethics state that animals will be excluded from studies if they display any symptoms of ill health affecting "activity, posture, appetite, behaviour, response to carers and weight maintenance".
Replication	To verify the reproducibility of these findings: All in vitro experiments were conducted 2 or more times. In vivo efficacy was tested in three different DIPG tumor models. All attempts at replication were successful.
Randomization	Animals were assigned to various experimental groups at random.
Blinding	The nature of the intervention meant that blinding was not possible, however each endpoint was confirmed by an independent observer.

Reporting for specific materials, systems and methods

We require information from authors about some types of materials, experimental systems and methods used in many studies. Here, indicate whether each material, system or method listed is relevant to your study. If you are not sure if a list item applies to your research, read the appropriate section before selecting a response.

Materials & experimental systems

n/a	Involved in the study	
☐	☒	Antibodies
☐	☒	Eukaryotic cell lines
☒	☐	Palaeontology
☐	☒	Animals and other organisms
☒	☐	Human research participants
☐	☒	Clinical data

Methods

n/a	Involved in the study	
☒	☐	ChIP-seq
☐	☒	Flow cytometry
☒	☐	MRI-based neuroimaging

Antibodies

Antibodies used

ODC1 (anti-ornithine decarboxylase): Abcam, ab97395.
SLC3A2 (anti-CD98): Bio-RAD, VPA00372.
Cleaved PARP: Cell Signalling Technology, Asp214.
Caspase 8: Cell Signalling Technology, D35G2.
Phospho-Histone H2A.X: Cell Signalling Technology, 9718.
p-m-TOR: Cell Signalling Technology,2971S.
m-TOR: Cell Signalling Technology, 2983.
p-4EBP1: Cell Signalling Technology, 236B4.
4EBP1: Cell Signalling Technology, 9644S.
GAPDH: Cell Signalling Technology, 14C10.
Actin: Cell Signalling Technology, D6A8.
Anti-Rabbit IgG HRP-linked antibody #7074: secondary antibody.
Anti-Mouse IgG HRP-linked antibody #7076: secondary antibody.

Validation

ODC1 (anti-ornithine decarboxylase): validated for human in WB.
SLC3A2 (anti-CD98): validated for human in WB.
Cleaved PARP: validated for human in WB.
Caspase 8: validated for human in WB.
Phospho-Histone H2A.X: validated for human in WB.

p-m-TOR: validated for human in WB.
m-TOR: validated for human in WB.
p-4EBP1: validated for human in WB.
4EBP1: validated for human in WB.
GAPDH: validated for human in WB.
Actin: validated for human in WB.

Eukaryotic cell lines

Policy information about cell lines

Cell line source(s)	Patient--derived primary cultures developed by the authors and collaborators. MRC5: obtained from ATCC and cultured according to manufacturer's instructions.<br>NHA: obtained from Lonza and were grown according to manufacturer's instructions.<br>SU-DIPGVI, SU-DIPGXVII: A/Prof. Michelle Monje, Stanford University.<br>HSJD-DIPG007,HSJD-DIPG008, HSJD-DIPG011, HSJD-DIPG012, HSJD-DIPG013: Dr. Angel Montero Carcaboso, Hospital Sant Joan de Deu.<br>VUMC-DIPG10: Dr. Esther Hulleman, Prinses Maxima Centrum.<br>RA055, RA038,P000302: developed from ZCC/PRISM clinical trial.
Authentication	STR profiling.
Mycoplasma contamination	All cell lines tested negative for mycoplasma contamination.
Commonly misidentified lines<br>(See ICLAC register)	No commonly misidentified lines were used.

Animals and other organisms

Policy information about studies involving animals; ARRIVE guidelines recommended for reporting animal research

Laboratory animals	Pathogen-free, 5-7 week old female Balb/C nude mice were purchased from Animal Resources Centre (Perth, Australia) and kept at an ambient temperature of 18-22° C with a humidity of 45-65% under a 12-hour light cycle (7:00 am -7:00pm).
Wild animals	The study did not involve wild animals.
Field-collected samples	The study did not involve samples collected form the field.
Ethics oversight	All experiments were performed under approval by the Animal Use and Care Committees of University of New South Wales.

Note that full information on the approval of the study protocol must also be provided in the manuscript.

Clinical data

Policy information about clinical studies

All manuscripts should comply with the ICMJE guidelines for publication of clinical research and a completed CONSORT checklist must be included with all submissions.

Clinical trial registration	This is a multicentre prospective study of the feasibility and clinical value of a diagnostic service for identifying therapeutic targets and recommending personalised treatment for children and adolescents with high-risk cancer. ClinicalTrials.gov number is NCT03336931.
Study protocol	The full trial protocol is not publically accessible as per our standard procedure. However, a detailed overview of the trial is available on ClinicalTrials.gov.
Data collection	Clinical data is collected at study enrolment and every 3 months while on study. Patients are followed up for 5 years from the time of enrolment or until death, whichever is sooner. All required clinical data is entered into LabMatrixTM, a laboratory and research information management system.
Outcomes	Primary outcome measure:<br>1. Personalised medicine recommendation<br>o Proportion of patients for whom personalised medicine recommendation can be made using a comprehensive diagnostic platform within a clinically relevant timeframe<br><br>Secondary outcome measures:<br>1. Tumour samples with actionable molecular alterations<br>o Proportion of tumour samples found to have actionable molecular alterations<br><br>2. Successfully conducted in vitro high throughput drug screening and in vivo drug sensitivity testing<br>o Proportion of tumours where in vitro high throughput drug screening and in vivo drug sensitivity testing can be successfully performed<br><br>3. Identification of potential treatment by in vitro or in vivo drug screening<br>o Proportion of tumours for which a potential treatment option is identified by in vitro or in vivo drug screening<br><br>4. Reporting turnaround time<br>o Number of weeks from enrolment to issuing a report to the treating clinician<br><br>5. Patients receiving the recommended personalised therapy<br>o Proportion of patients who subsequently receive the recommended personalised therapy<br><br>6. Barriers or reasons for patients not receiving the recommended personalised therapy<br>o Description of the barriers or reasons for patients not receiving the recommended personalised therapy

Flow Cytometry

Plots

Confirm that:

- [x] The axis labels state the marker and fluorochrome used (e.g. CD4-FITC).
- [x] The axis scales are clearly visible. Include numbers along axes only for bottom left plot of group (a 'group' is an analysis of identical markers).
- [x] All plots are contour plots with outliers or pseudocolor plots.
- [x] A numerical value for number of cells or percentage (with statistics) is provided.

Methodology

Sample preparation	Cultured cell lines as indicated were harvested and stained with relevant fluorochome conjugated antibodies.
Instrument	BD Canto analyzer.
Software	BD FacsDiva.
Cell population abundance	N/A
Gating strategy	Gating was performed using FSC/SSC. Apoptotic population in each treatment cohort was determined by combining the Annexin V-FITC positive and the 7AAD/Annexin V-FITC positive populations.

- [x] Tick this box to confirm that a figure exemplifying the gating strategy is provided in the Supplementary Information.
